# Modularized supramolecular assemblies for hypoxia-activatable fluorescent visualization and image-guided theranostics

**DOI:** 10.7150/thno.95590

**Published:** 2024-06-11

**Authors:** Wen Liu, Bincheng Wang, Bei Guo, Junbin Zhu, Zejun Xu, Jiayue Xu, Zhen Wang, Guodong Sun, Wei Wang, Yi Zhang, Wei Xue

**Affiliations:** 1 Engineering Technology Research Center of Drug Carrier of Guangdong, Department of Biomedical Engineering, Jinan University, Guangzhou 510632, China.; 2 China Guangdong Provincial Key Laboratory of Spine and Spinal Cord Reconstruction, The Fifth Affiliated Hospital (Heyuan Shenhe People's Hospital), Jinan University, Heyuan 517000, China.; 3 College of Pharmacy, Jinan University, Guangzhou 510630, China.; 4 Guangdong Provincial Key Laboratory of Optical Fiber Sensing and Communication, Institute of Photonics Technology, Jinan University, Guangzhou 510632, China.; 5 Department of Orthopedics, The First Affiliated Hospital, Jinan University, Guangzhou 510630, China.; 6 Bai Yun Shan Pharmaceutical General Factory, Guangzhou Bai Yun Shan Pharmaceutical Holdings Co. Ltd. Guangzhou 510515, China.

**Keywords:** Tumor heterogeneity, hypoxia, supramolecular assembly, fluorescent probe, photodynamic therapy

## Abstract

**Rationale**: Molecular imaging of microenvironment by hypoxia-activatable fluorescence probes has emerged as an attractive approach to tumor diagnosis and image-guided treatment. Difficulties remain in its translational applications due to hypoxia heterogeneity in tumor microenvironments, making it challenging to image hypoxia as a reliable proxy of tumor distribution.

**Methods**: We report a modularized theranostics platform to fluorescently visualize hypoxia via light-modulated signal compensation to overcome tumor heterogeneity, thereby serving as a diagnostic tool for image-guided surgical resection and photodynamic therapy. Specifically, the platform integrating dual modules of fluorescence indicator and photodynamic moderator using supramolecular host-guest self-assembly, which operates cooperatively as a cascaded “AND” logic gate. First, tumor enrichment and specific fluorescence turn-on in hypoxic regions were accessible via tumor receptors and cascaded microenvironment signals as simultaneous inputs of the “AND” gate. Second, image guidance by a lighted fluorescence module and light-mediated endogenous oxygen consumption of a photodynamic module as dual inputs of “AND” gate collaboratively enabled light-modulated signal compensation *in situ*, indicating homogeneity of enhanced hypoxia-related fluorescence signals throughout a tumor.

**Results:** In *in vitro* and *in vivo* analyses, the biocompatible platform demonstrated several strengths including a capacity for dual tumor targeting to progressively facilitate specific fluorescence turn-on, selective signal compensation, imaging-time window extension conducive to precise normalized image-guided treatment, and the functionality of tumor glutathione depletion to improve photodynamic efficacy.

**Conclusion:** The hypoxia-activatable, image-guided theranostic platform demonstrated excellent potential for overcoming hypoxia heterogeneity in tumors.

## Introduction

Tumors encompass different cell subsets with variable genomes, phenotypes, molecular characteristics and functions. Such cellular and molecular diversity impacts the processes of tumor onset and evolution, generating tumor heterogeneity 1-3. Different levels of tumor heterogeneity exist among patients, between primary tumors and their metastases, and even within a single tumor with dynamic temporal and spatial microenvironment factors (such as extracellular matrix, immune cells, pH, oxygen tension, etc.) 4-8. In particular, hypoxia heterogeneity is pervasive in tumors and influences glycolysis, vascularization, stem cell phenotype, metastasis, and chemoradiation sensitivity 9-12. Consequently, random sampling and analysis of hypoxia tension regions do not accurately reflect tumor phenotypes or degrees of hypoxia, thus contributing to diagnostic errors and even failures of targeted therapy 13-16.

In recent years, exciting advances have been made in imaging tools for improved anti-tumor diagnosis and therapies. Hypoxia-activated fluorescence probes provide productive avenues for delineating spatial distribution of hypoxic regions around solid tumors 17-23. Under hypoxic conditions, for example, endogenous nitroreductases (NTRs) are overexpressed, which can be rationally exploited to catalyze the intracellular transformation of quenched fluorescence probes into a turn-on state for imaging or detection of hypoxia tension regions 24-28. Thus far, most tumor microenvironment-activated fluorescent probes have failed to pass clinical trials, due to a number of unresolved limitations including low delivery efficiency, inability to authentically report intact tumor distribution owing to heterogeneity, and restricted imaging window durations induced by rapid clearance. In addition, fluorescence intensities of activatable fluorescent probes typically depend on endogenous physiological signals which generally cannot be manipulated exogenously. Significantly, *in vivo* fluorescent signal quality is broadly influenced by tumor heterogeneity, identified as one of the most significant barriers to fluorescent imaging-guided diagnosis and treatment.

With the development of nanoscale drug delivery systems endowed with intelligent responsiveness to microenvironment physiological characteristics (such as pH, ROS, hypoxia, enzymes, etc.) for tumor treatment modalities, emphasis is on improving the tumor space anchoring of payloads 29-31. Nanocarriers perform well in integrating several functional modules to achieve extended blood circulation, tumor targeting, *in situ* degradation release, and in combining novel imaging technologies with therapeutic options 32. However, each module in the assembled nanocarriers often performs its intended molecular tasks individually, sometimes even functionally contradicting other modules 33. For instance, Chlorin e6 as a treatment module for photodynamic therapy (PDT), emits fluorescence, interfering with the fluorescence-based diagnostic module. Hence, it remains challenging to design new delivery systems whereby functional modules have collaborative and not divergent molecular tasks 34-38.

**Scheme 1:**
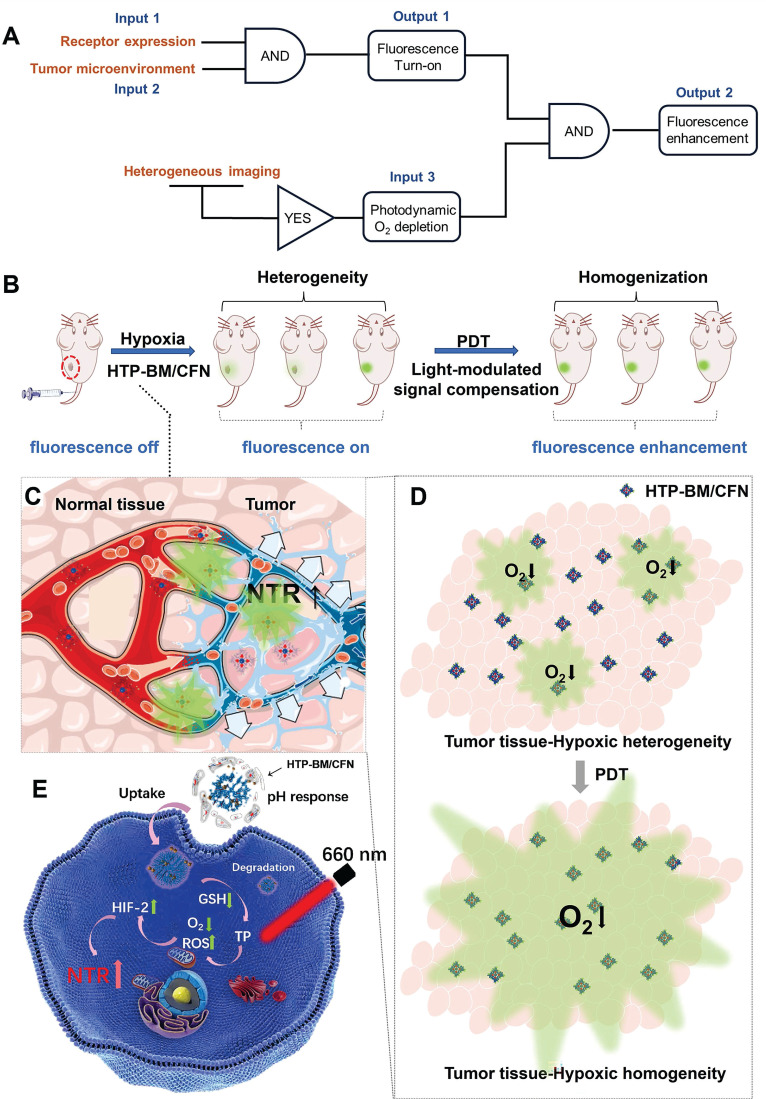
(A) Schematic illustration of the enhanced fluorescence imaging based on HTP-BM/CFN in terms of manner as two-stage “AND” logic gate. (B). Schematic illustration of the multifunctional assemblies (HTP-BM/CFN) as fluorescent indicators of hypoxia, as well as photosensitizers that normalize tumor hypoxic heterogeneity: (C). Overexpressed folate receptors recruit folate-decorated HTP-BM/CFN to be enriched at tumor sites, followed by disassembly of the shell layers, release of the fluorescent indicator (CNP) and photosensitizer. The CNP is turned on under hypoxia-induced NTR high expression. (D). Heterogeneous fluorescence feedback appears within a single tumor with spatial heterogeneity of hypoxic regions. After HTP-BM core in GSH induced dissociation state exert a phototherapeutic effect upon the laser treatment, the hypoxia is enhanced with expanded hypoxic region regulated by PDT process, stimulating an enhanced and homogenized trend of fluorescence over the whole tumor. (E). Enhanced PDT efficiency with imaging monitoring was achieved by depleting GSH and elevating ROS production during cellular HTP degradation together with up-regulation of NTR.

To this end, we conceived a modularly assembled hypoxia-activatable fluorescence theranostic agent capable of light-modulated signal compensation and performing collaborative molecular tasks as a two-stage “AND” logic gate functional module (Scheme 1A). At the first stage, tumor-specific fluorescence imaging turn-on and enrichment is an output with the dual actions of tumor microenvironment (characterized by hypoxia, low pH, and high glutathione (GSH) levels) and folate receptor as an input, where fluorescence imaging is enabled by activities of NTR highly expressed in hypoxic regions. In the second stage, the hypoxia-activated fluorescence module and photodynamic module-induced oxygen consumption collaboratively serve as inputs to enable light-modulated tumor fluorescence compensation *in situ* as the output of “AND” logic gate. Consequently, the cascaded “AND” logic gate of this theranostic agent improves diagnosis enabling an enhanced, even fluorescence distribution throughout the imaged tumors, conducive to the ensuing de-heterogeneization of hypoxia fluorescence imaging (Scheme 1B).

**Scheme 2:**
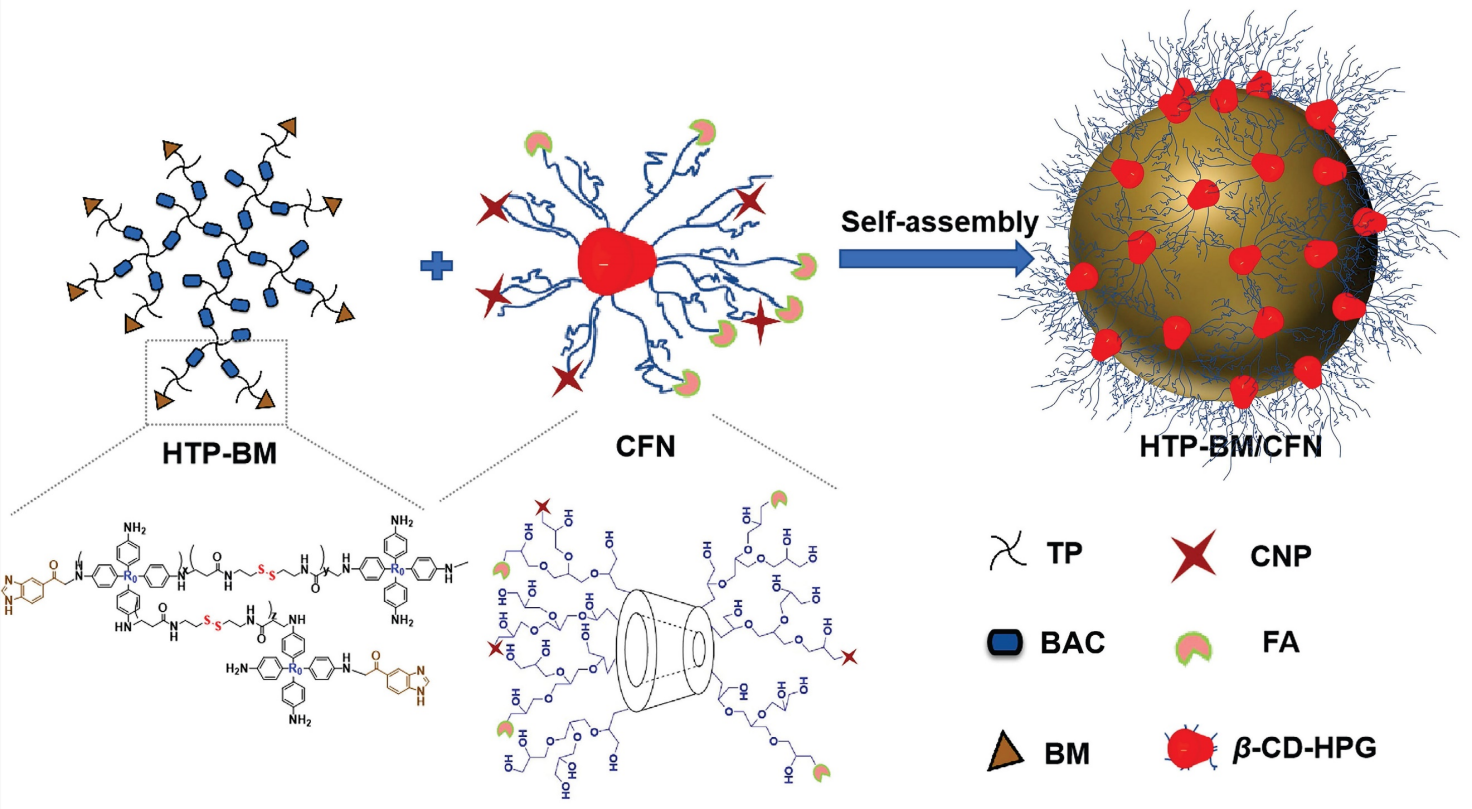
Self-assembly diagram of HTP-BM/CFN assemblies via host-guest interaction, and the corresponding chemically structures of each component and functionality.

Our modularized theranostic agent is assembled from a core of benzimidazole-modified polyporphyrin (HTP-BM) rich in disulfide bonds, a shell (CFN) composed of a hyperbranched glycidyl ether cyclodextrin (*β*-CD-HPG) modified with folic acid (FA) and a hypoxia-activatable fluorescent probe (CNP) (Scheme 2). The host-guest supramolecular self-assembly of benzimidazole and cyclodextrin leads to the formation of a hierarchical core-shell structure, which can be deconstructed in the weakly acidic tumor microenvironment 39. The HTP-BM/CFN assemblies combine active (FA receptor) and passive targeting (*in situ* disassembly in weakly acidic microenvironments and GSH-triggered HTP degradation) to achieve enrichment of the photosensitizer HTP and the fluorescent probe CNP as tumor-specific spatial anchors (Scheme 1C). Consequently, oxygen consumption of polyporphyrin-based PDT modulated by external light irradiation is targeted to tumor regions, while the hypoxia threshold of the tumor is tuned and raised. The results of this amplification and de-heterogenization can be realized by hypoxia fluorescent probes, resolving hypoxia heterogeneity and extending the time window in imaging (Scheme 1D). Thus, enhanced PDT with visual monitoring was accomplished by depleting cellular GSH and elevating ROS production during HTP degradation coupled with light modulation (Scheme 1E). Therefore, HTP-BM/CFN as a diagnosis and treatment platform can be exploited in highly specific fluorescent imaging-guided tumor therapy. In summary, by using multiple tumor intrinsic attributes as spatial anchor targets, we demonstrated an innovative strategy to overcome the challenges of hypoxia heterogeneity and improve hypoxia fluorescence imaging and therapeutic effects.

## Results and Discussion

### Preparation and characterization of HTP-BM/CFN assemblies

The HTP-BM/CFN theranostic platform was prepared by self-assembly of the shell (CFN) and core (HTP-BM), through supramolecular host-guest interaction between BM and *β*-cyclodextrin (Scheme 2). For the synthesis of HTP-BM core (as illustrated in Scheme S1), N, N'-bis(acryloyl) cystamine (BAC) was first synthesized by an addition reaction between acryloyl chloride and cystamine, followed by the synthesis of hyperbranched polyporphyrin (HTP) incorporated with disulfide bonds via a Michael addition reaction between 5,10,15,20-tetra (4-aminophenyl) porphyrin (TP) and BAC. Finally, a hyperbranched polymer with benzimidazole capping (HTP-BM) was prepared by the amidation reaction. As shown in Scheme S2, the CFN shell was obtained by a simple esterification reaction of grafting FA and CNP onto, the host molecule *β*-CD-HPG, which was synthesized by a ring-opening polymerization reaction of glycidol 40-43. The NTR-sensitive fluorescent probe CNP was synthesized by a five-step procedure (Scheme S3), and a carboxylic acid group was introduced for subsequent esterification reaction with *β*-CD-HPG.

**Figure 1:**
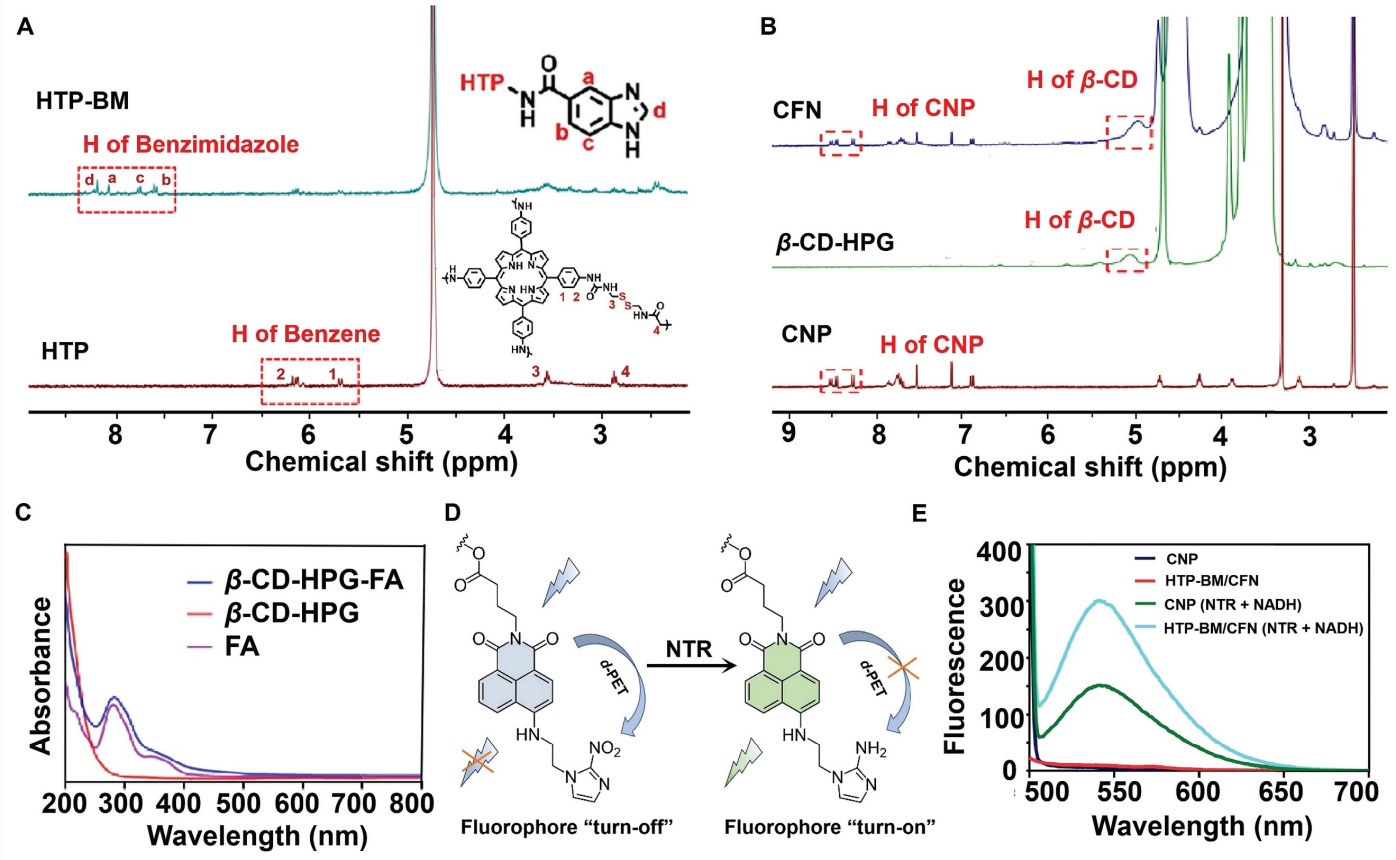
Characterization of core (HTP-BM) and shell (CFN) molecules. (A) ^1^H NMR spectra of HTP-BM. and HTP. (B) ^1^H NMR spectra of CFN, *β*-CD-HPG and CNP. (C) The comparison results of UV absorption spectra between FA, *β*-CD-HPG and CFN. (D) Fluorescence turn-on mechanism of the fluorophore CNP. (E) Fluorescence emission spectrum of CNP, HTP-BM/CFN, CNP (NTR+NADH) and HTP-BM/CFN (NTR + NADH) (λ_ex_ = 436 nm).

To validate the co-polymerization of TP and BAC to form the HTP core, we characterized and compared the molecules before and after the polymerization reaction by UV and IR absorption spectra (Figure S1A). BAC gave no absorption peaks in the visible light spectral region, while the characteristic peak of the porphyrin (TP) molecule appeared at 436 nm for HTP. IR spectra (Figure S1B) showed characteristic peaks of both -C-S- (near 2,837 cm^-1^) belonging to BAC and -C=N- (1690 cm^-1^) attributed to the presence of several pyrrole rings in the porphyrin ring, reflecting the successful polymerization of the two monomers (TP and BAC) to yield HTP. Additionally, benzimidazole was attached to HTP by the amidation reaction to form HTP-BM and was subjected to ^1^H NMR analysis (Figure 1A). The ^1^H NMR spectra of the HTP compound at H_4_ 2.92 ppm (t, 4H, CONHCH_2_CH_2_S) and H_3_ 3.52 ppm (t, 4H, CONHCH_2_CH_2_S) represented hydrogen proton peaks on CH_2_ attributed to cystamine while the two cleavage peaks at 5.5-6.4 ppm were the CH peaks on the benzene ring of HTP. By integrating characteristic peaks, the molar content of TP in the compound was estimated to be about 23.6%. The coupling of benzimidazole on HTP was corroborated by the four cleavage peaks located between 7.5 and 8.2, which were attributed to the characteristic peaks Ha~Hd on the imidazole ring (Figure 1A).

Chemical structures of the NTR-activatable fluorescent probe (CNP) and its intermediates were characterized by ^1^H NMR spectroscopy, ^13^C NMR spectroscopy, and high-resolution mass spectrum spectrometry (Figures S2-S13). We characterized the chemical structure of the shell molecule CFN by ^1^H NMR to validate the successful grafting of the CNP probe and folate molecule onto the hyperbranched polyglycerol modified *β*-cyclodextrin (*β*-CD-HPG). As shown in Figure 1B, the hydrogen peaks at chemical shifts of 7-8.5 ppm in the CFN spectra were attributed to characteristic benzene ring peaks on the CNP fluorophore. FA has a UV absorption peak at around 280 nm, while *β*-CD-HPG has no characteristic UV absorption peak at this wavelength. We verified the successful grafting of FA onto shell molecules by comparing the UV absorption peaks of FA and CFN, and the grafting rates of CNP and FA on the *β*-CD-HPG shell molecules were estimated to be about 5.93 % and 3.7 %, respectively (Figure 1C).

Under hypoxic conditions, the NTR was highly expressed in the tumor cells. The probe CNP contained a molecular recognition unit (2-nitroimidazole) for detecting the NTR activity (CNP displayed high selectivity for NTR, strong fluorescence enhancement (108 folds), and a low detection limit (3.6 ng/mL)). NTR mediated the transformation of nitro to an amino group on the recognition unit of CNP, thereby disabling photo-induced electron transfer (d-PET), as illustrated in Figure 1D. Conjugation with probe CNP confers the same imaging performance of the CFN shell molecules to NTR. In fluorescence spectrum experiments, following the addition of NTR and nicotinamide adenine dinucleotide (NADH) to simulate a hypoxic environment, HTP-BM/CFN exhibited a fluorescence "turn-on" effect comparable to CNP probes (Figure 1E). In contrast, its fluorescence emission at 536 nm was not hindered by other structures in the assembly system.

**Figure 2:**
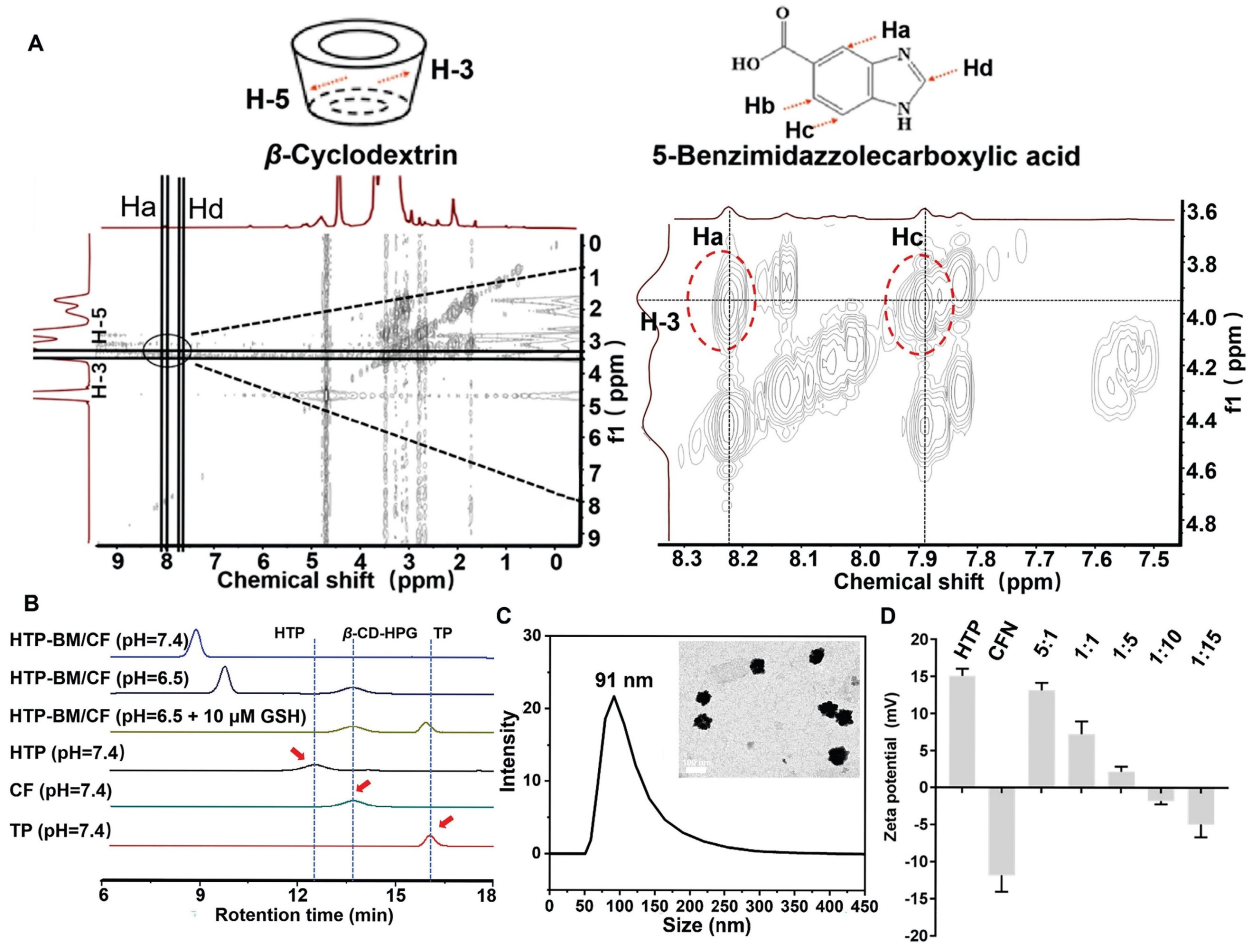
Characterization of the HTP-BM/CFN core-shell self-assembly. (A) Two-dimensional NMR spectra of the HTP-BM/CFN assemblies. (B) GPC traces of TP, CF, HTP, and HTP-BM/CF under different treatment conditions. (C) Particle size distribution and morphology characterization of the assemblies (scale bar = 100 nm). (D) Zeta potential of the assemblies with different core/shell ratios.

The core-shell self-assembly behavior of HTP-BM/CFN was validated by 2D-NOESY spectroscopy to confirm the successful acquisition of host-guest assembly. A locally enlarged contour 2D-NOESY diagram (Figure 2A) shows that the H-3/H-5 proton inside the hydrophobic cavity of *β*-cyclodextrin (host) and the aromatic Ha/Hc proton of benzimidazole residue (guest) between 8.22 and 7.89 resonance of the four cleavage peaks had a significant correlation. The proximity between these protons confirmed the formation of a self-assembled structure.

Next, we validated the responsiveness of core-shell assemblies in weakly acidic and GSH-rich tumor microenvironments, by simulating tumor-like conditions with low pH and high GSH concentration *in vitro*. The pH-dependent disassembly and GSH-dependent degradation behavior of HTP-BM/CF were characterized by gel permeation chromatography (GPC). The retention time peaks on GPC represented the molecular weights of different samples (Figure 2B). The fragment molecules HTP and CF with molecular weights 4,759 and 3,586, respectively, served as references. When all other sample groups were kept at pH = 7.4, the retention time of HTP and CF became 12.7 min and 13.6 min, respectively. The retention time of the HTP-BM/CF assemblies was shifted to 8.7 min, suggesting that the formation of core-shell assembly between the host and guest molecules, resulted in higher molecular weights. However, at pH = 6.5, the HTP-BM/CF peak with a delayed efflux time (~9.5 min) was observed, indicating its disassembly of HTP-BM/CF under the tumor acidic microenvironment. Moreover, extended retention peaks were found at 13.6 (CF) and 16.5 min (TP) in the presence of low pH and high GSH, implying further GSH-induced HTP degradation, thereby leading to a drop in macromolecular weight, which is conducive to metabolic clearance. Besides, the decomposition of HTP-BM/CF under normal pH and high GSH concentration was also measured (Figure S14). The results confirmed that at low pH and high GSH, typical of tumor microenvironment, there was sequential HTP-BM/CF disassembly and HTP degradation.

The size and morphology of HTP-BM/CFN were further characterized by transmission electron morphology (TEM). As displayed in Figure 2C, the morphology of the assemblies was spherical with a mean diameter of 89.57 ± 9.7 nm. Similar to TEM, dynamic light scattering (DLS) analysis further showed that the average particle size was 91 ± 14.7 nm (Figure 2C) with a broadly ideal size distribution (average polydispersity index less than 0.2). The stability of the HTP-BM/CFN assembly was also tested, showing good stability under physiological conditions, but forming large aggregates under a simulated tumor acidic microenvironment (Figure S15-S16). Furthermore, the zeta potential measurements revealed that the HTP-BM and CFN surface charges were 15 ± 1.71 mV and -12.1 ± 2.23 mV, respectively. Surface potentials were neutralized as the ratio of core-shell molecules was adjusted during the assembly process (Figure 2D). The surface charge of the assemblies was -5.21 ± 0.38 mV when the core-shell assembly quality ratio (core: shell) was 1:10, which was chosen for subsequent experiments. Collectively, these results suggested that the core-shell assembly plays a significant role in surface charge regulation, as negatively charged delivery systems can prevent clearance during blood circulation.

### Cellular internalization and tumor targeting behavior of HTP-BM/CFN

**Figure 3:**
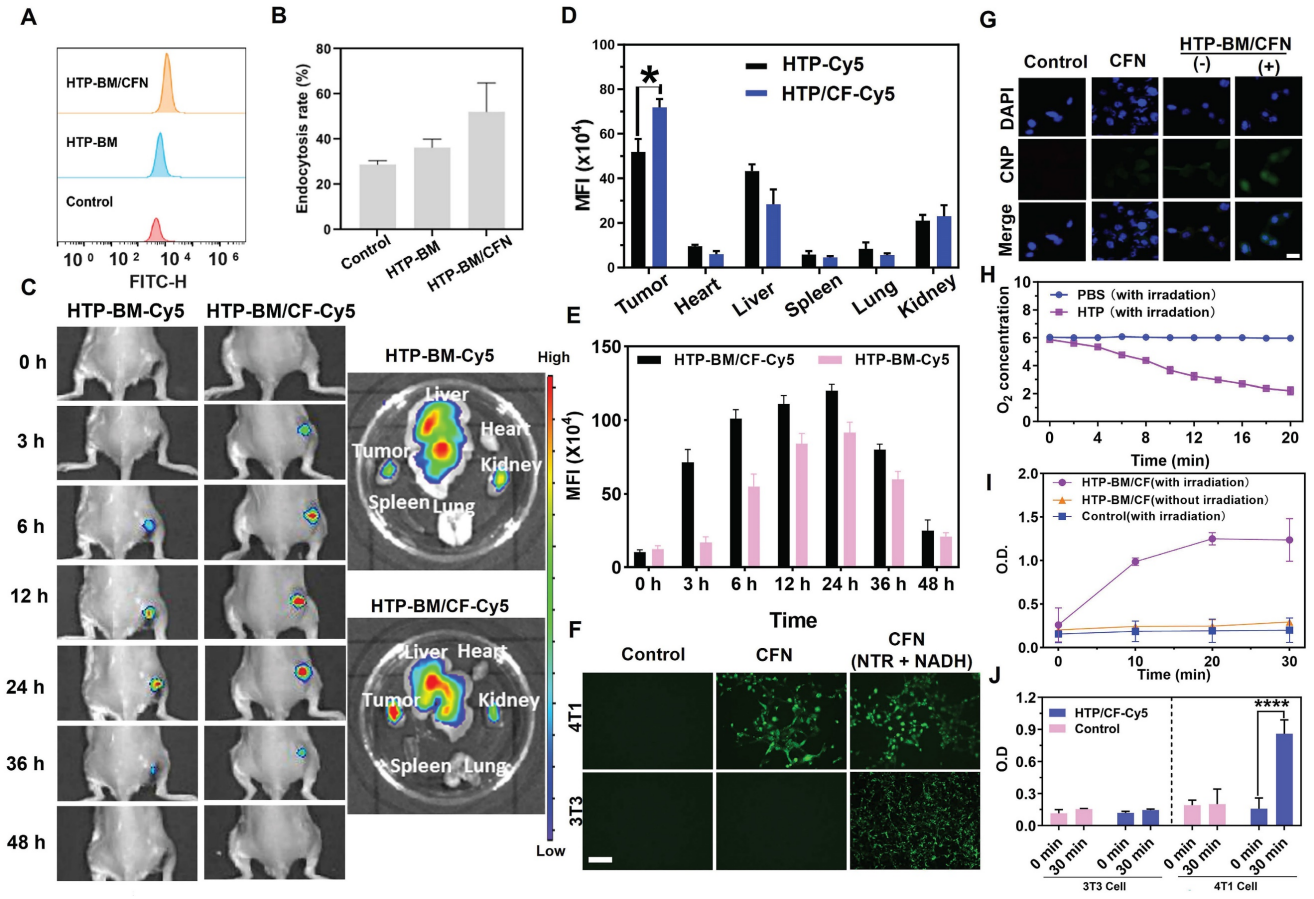
(A) Flow cytometric analysis of HTP-BM and HTP-BM/CF uptake by 4T1 cells and quantitative fluorescence statistics (B). (C) *In vivo* fluorescence imaging of HTP-BM-Cy5 and HTP-BM/CF-Cy5 in mice and ex-vivo isolated tumors and major organs 48 h after tail vein injection. Quantitative fluorescence statistics of *in vivo* imaging in major organs (D) and tumor along the time (E) in mice. (F) Fluorescence images of 4T1 and 3T3 cells co-incubated with PBS, CFN and CFN (10 μL NTR, 2 μL NADH) for 6 h, respectively (scale bar is 100 μm). G) Fluorescence images of 4T1 cells incubated with CFN and HTP-BM/CFN with or without 10 min laser irradiation (660 nm, 1.2 W/cm^2^), (-) representing without irradiation, (+) representing with irradiation, scale bar: 20 μm. (H) Dissolved oxygen content in PBS and HTP solutions before and after laser irradiation (660 nm, 1.2 W/cm2). (I) Nitroreductase activity in 4T1 cells in the presence and absence of HTP-BM/CF after irradiation treatment with the time. (J) Nitroreductase activity in 4T1 cells and 3T3 cells in the presence and absence of HTP-BM/CF before and after 30 min of irradiation treatment. (* *p*<0.05, and **** *p*<0.0001)

We investigated the cellular internalization of HTP-BM/CFN, by incubating HTP and HTP-BM/CFN (equivalent porphyrin, and Cy 5.5 labeling) with 4T1 cells for a fixed time interval. Cellular uptake was analyzed by flow cytometry (Figure 3A). HTP and HTP-BM/CFN could enter the cells effectively through endocytosis. Figure 3B shows that the fluorescence signal in the HTP-BM/CFN-treated group was significantly enhanced, and the endocytosis rate reached 52.4 %, compared with 35.6 % in the HTP-treated cells, demonstrating that the assemblies with targeted folate-grafted shells could be easily internalized into the tumor cells.

Enrichment of the theranostic agents at the tumor site directly enhances their imaging quality and therapeutic effects *in vivo*. Thus, we investigated the *in vivo* tumor targeting ability of the core-shell assembled HTP-BM/CF compared with HTP-BM in mice. HTP-BM/CF assemblies were enriched at the tumor site of mice 3 h after the tail vein injection (Figure 3C), while no fluorescence signal was observed at the tumor site with HTP-BM at the same time point. When measured between 6-24 h, the fluorescence signal at the tumor site in the HTP-BM/CF-injected mice was significantly higher than in mice receiving HTP-BM (Figure 3D), which could be due to targeting effects mediated by the folate ligand, disassembly of the core shell triggered by the tumor microenvironment, and the long circulation ability of the polyglycerol ether shell. Subsequently, the mice were euthanized, and major organs and tumors were collected to determine the accumulation of fluorescence (Figure 3C-E). A higher tumor accumulation of the core-shell-assembled HTP-BM/CF than HTP-BM alone was observed, indicating superior *in vivo* tumor targeting of the theranostic agent assembled with the CF shell.

### *In vitro* fluorescence turn-on and light-modulated signal enhancement by HTP-BM/CFN

To simulate turn-on effects of the CNP fluorescent probe in the hypoxic and NTR rich microenvironment of tumors, we investigated the *in vitro* turn-on effects of HTP-BM/CFN in tumor cells incubated in a sealed bag pretreated with anoxic gas-generation (Figure 3F). Significant green fluorescence could be observed when 4T1 cells were incubated with HTP-BM/CFN for 6 h, In contrast, fluorescence was weak following incubation of 3T3 cells with HTP-BM/CFN under the same conditions. NTR-sensitive turn-on effects of CFN were confirmed by the appearance of bright fluorescence in 4T1 and 3T3 cells, when CFN was employed as the positive control in the presence of NTR and NADH. These results suggested that the hypoxic microenvironment of tumor cells could induce high expression of NTR, triggering CNP fluorescence incorporated in HTP-BM/CFN, raising the possibility of tumor-specific hypoxia imaging *in vivo*.

After determining the tumor microenvironment-responsive fluorescence turn-on effect of HTP-BM/CFN, we used 4T1 cells to further investigate the ability to modulate the fluorescence signal amplification by photodynamic oxygen depletion at the cellular level. As shown in Figure 3G, laser irradiation treatment significantly enhanced the fluorescence signal in the HTP-BM/CFN-treated cells compared to cells without laser irradiation. We speculated that the fluorescence signal enhancement of HTP-BM/CFN under laser irradiation could be due to oxygen depletion caused by the HTP core-mediated photodynamic process, leading to hypoxia within the tumor cells and thereby upregulating the intracellular NTR expression. Hence, it indicated that this light-modulated fluorescence compensation technique based on HTP-BM/CFN could enhance the endogenous fluorescence signal intensity by spatially controllable external light irradiation, offering an opportunity to regulate *in vivo* imaging signal by exogenous intervention.

Utilizing O_2_ as a precursor is a hallmark event in the PDT process 44. The oxygen-consuming capacity of HTP was compared by dissolving oxygen in PBS solution with and without HTP under 20-minute laser irradiation. Figure 3H shows that the amount of dissolved oxygen was significantly reduced in the HTP-containing PBS under irradiation, indicating the significant oxygen consumption ability of HTP within the assembly.

To further verify the upregulation of NTR in tumor cells after oxygen depletion caused by the HTP-induced photodynamic process, the intracellular NTR activity was measured in the presence of HTP-BM/CF with and without laser irradiation (660 nm, 1.2 W/cm^2^). As shown in Figure 3I, NTR activity in 4T1 cells was significantly increased over time in the presence of HTP-BM/CF after irradiation, but not altered in cells without light irradiation. Thus, enhanced NTR level and activity were related to the hypoxic tumor microenvironment induced by HTP-mediated photodynamic processes. Our results were consistent with previous studies reporting that hypoxia accelerates biological reduction reactions leading to the overexpression of intracellular reductases (e.g. DT-cardiac flavonase, azoreductase, and nitroreductase), which serve as an important indicator of the hypoxic state of tissues and organisms 45.

Our results showed that NTR content varied in different cells and was higher in tumor cells (4T1) than in normal cells (3T3) (Figure 3J). Interestingly, following laser irradiation of HTP-BM/CF, the NTR content in 4T1 cells was notably higher than in 3T3 cells. This phenomenon could also be attributable to faster oxygen consumption of tumor cells, aggravating the hypoxic microenvironment to promote NTR expression. Another reason could be that there was a selective enrichment of HTP-BM/CF in tumor cells due to folic acid receptor-mediated endocytosis.

### *In vivo* tumor-specific fluorescence imaging and light-modulated signal enhancement by HTP-BM/CFN

**Figure 4:**
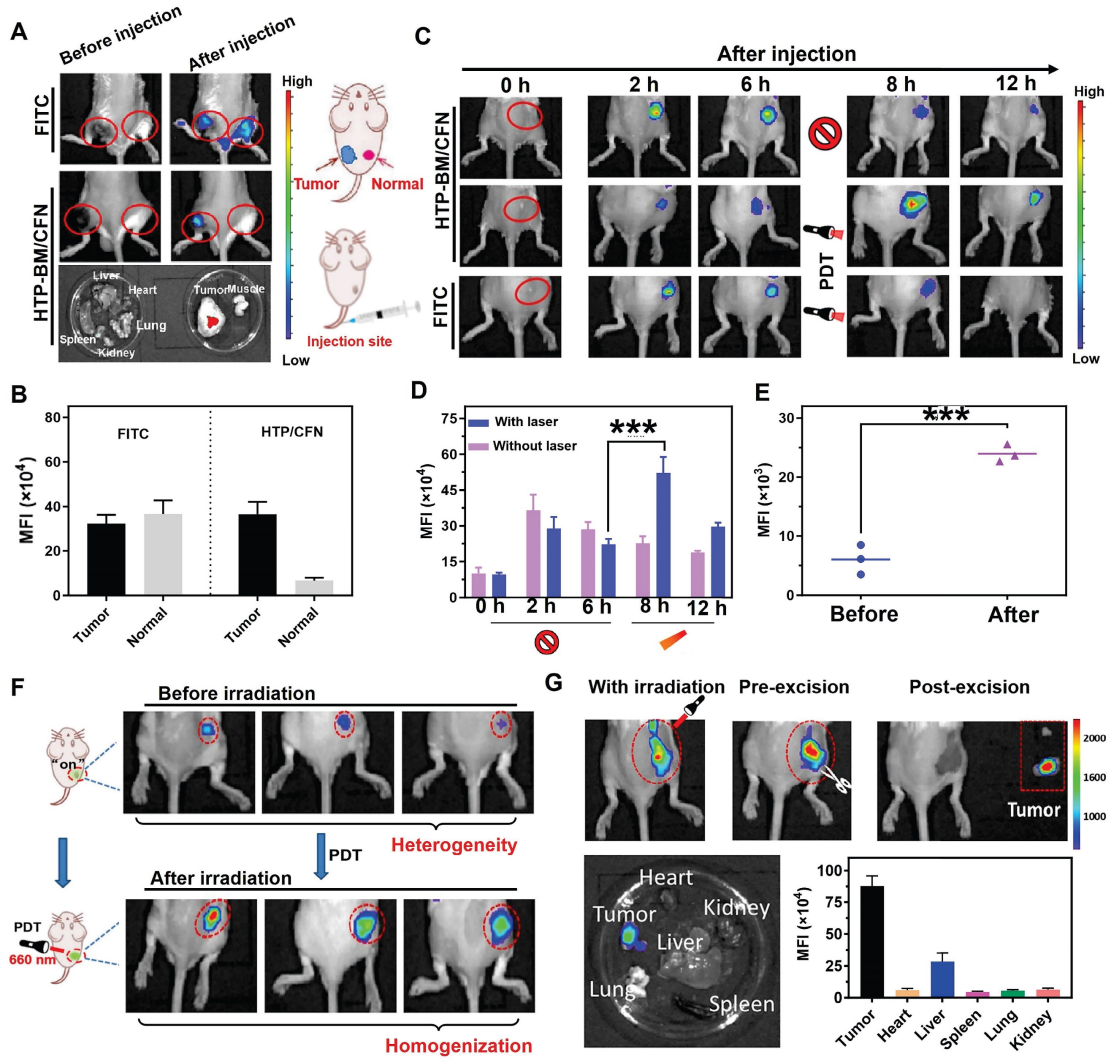
(A) *In vivo* fluorescence imaging of tumor and muscle tissues before and after injected with FITC and HTP-BM/CFN respectively in mice and their fluorescent quantitative analysis (B). (C) *In vivo* fluorescence image along the time (0-6 h) after administrated with FITC and HTP-BM/CFN respectively, followed by light irradiation at 6h for fluorescence enhancement and their fluorescence quantification(D). (E) fluorescence quantification. (F) *In vivo* fluorescence image of light-modulated heterogeneous tumor imaging. (G) Real-time fluorescent imaging-guided tumor surgical resection based on the light-modulated fluorescence compensation of HTP-BM/CFN and fluorescent quantitative analysis of main organs and tumor. (*** *p*<0.001)

We investigated whether HTP-BM/CFN assemblies could specifically visualize the tumor hypoxic microenvironment *in vivo* without generating false positive signals in normal tissues. For this, the HTP-BM/CFN and NTR-independent fluorescent dye FITC (control group) were simultaneously injected *in situ* into tumor tissues and normal muscle tissues of mice. *In vivo* fluorescence imaging of the two sites is shown in Figure 4A. Compared to the always-on fluorescent dye (FITC) that showed a similar fluorescence signal in both tumor and muscle sites, HTP-BM/CFN revealed specific fluorescence only in the tumor tissue. Also, the *ex vivo* fluorescence imaging of main organs and HTP-BM/CFN-injected tissues confirmed the tumor-specific fluorescence turn-on ability of HTP-BM/CFN. Additionally, the mean fluorescence intensity (MFI) of the HTP-BM/CFN assemblies was further quantified and was much higher in the tumor site than in the muscle tissue (Figure 4B), consistent with the *in vitro* results. These observations suggested that the HTP-BM/CFN, as a fluorescent imaging agent, can be accurately visualized at the tumor site without the false positives caused by the non-specific accumulation of conventional probes.

PDT-induced NTR upregulation experiments reinforced cell imaging of HTP-BM/CFN, indicating its potential as a turn-on fluorescence imaging agent capable of light-modulated fluorescence enhancement. Therefore, we observed fluorescence signal intensity over time in mice after intravenous injection of HTP-BM/CFN (Figure 4C-D). Fluorescence signals at the tumor site peaked at 6 h after HTP-BM/CFN injection and then faded by 8 and 12 h, suggesting that the time window for the *in vivo* imaging of the CNP probe was within 6 h. On the other hand, after performing (660 nm) laser irradiation of the region with fluorescence turn-on 6 h after HTP-BM/CFN injection, we found a 1.78-fold enhancement in the fluorescence intensity of the tumor region post-irradiation at 8 h, thereby extending the imaging time window to 12 h. This result was consistent with *in vitro* cell experiments. The fluorescence intensities in mice with intratumorally injected FITC did not show a significant increase in the tumor area following laser irradiation and gradually faded over time.

Our results showed that the light-modulated fluorescence compensation capacity of HTP-BM/CFN *in vivo* was beneficials for integrating the NTR-activated fluorescent probe (CNP) and photodynamic-triggered oxygen regulation (HTP) modules, leveraging hypoxia-induced NTR upregulation under physiological environment. Additionally, this technique of light-modulated fluorescence enhancement based on HTP-BM/CFN achieved external field-tuned internal physiological signal variation that facilitated fluorescence signal compensation *in vivo*, and extended the time window of fluorescence imaging conducive to different types of image-guided treatments.

### Light-modulated fluorescence compensation overcomes heterogeneity in tumor imaging

Tumor heterogeneity refers to the diverse subclones of tumor cells, stromal cells, and microenvironmental conditions that determine tumor growth, progression, and response to therapy 46-49. The degree and spatial heterogeneity of tumor hypoxia and related NTR expression usually differ among tumors. Due to the PDT-modulated fluorescence compensation, tumors with heterogeneous fluorescence could reach relatively homogeneous fluorescence levels *in vivo* following intravenous injection of HTP-BM/CFN (Figure 4F). Furthermore, quantitative results (Figure 4E) showed that the fluorescence intensity at tumor sites in mice increased following laser irradiation by 1.36 folds, attributable to the light-modulated hypoxia and NTR expression enhancement by the photodynamic process, Heterogeneity in fluorescence imaging derived from individual differences could be tuned to a relatively homogenous state by selecting different laser irradiation times or power.

Enhanced fluorescence imaging effects are determined by a combination of multiple functions of the HTP-BM/CFN assemblies. This collaborative mechanism is referred to as two-stage AND logic gate (Scheme 1D). First, fluorescence imaging turn-on AND logic gate with TME and receptor expression as inputs, included passive targeting induced by microenvironment physiological signals (e.g., hypoxia, low pH), and active targeting induced by high-expression receptors (e.g., folate). Second, enhanced fluorescence imaging was only achievable with the input of fluorescence imaging spatial guidance and photodynamic-triggered oxygen depletion that contributed to selective NTR upregulation. Essentially, such a two-stage AND logic gate design improved the accuracy of tumor-specific fluorescence imaging and the tunability of image-guided fluorescence enhancement.

### Enhanced fluorescence imaging-guided precise tumor surgical resection based on HTP-BM/CFN

While factors such as limited tumor specificity, heterogeneity and time window of fluorescent imaging are major obstacles to the current practice of fluorescence imaging-guided tumor resection, the HTP-BM/CFN with tumor-specific fluorescence turn-on and light-modulated signal enhancement may provide potential solutions. As shown in Figure 4G, the tumor region could be clearly located, while the boundary of tumor tissue could be intuitively identified with the guidance of HTP-BM/CFN-mediated fluorescent imaging *in vivo* following enhancement by proper photodynamic treatment. Subsequently, tumor resection was conducted and the lesion was removed entirely under the guidance of fluorescent imaging with extended time window. *Ex vivo* imaging indicated that the fluorescence signals were concentrated mainly in the tumor but not in the vital organs, verified by fluorescence quantitation. Using a two-stage AND logic gate design, this HTP-BM/CFN-based tumor-specific fluorescence imaging coordinated with the light-modulated fluorescence enhancement technique illustrated a novel approach to assisting in real-time surgery by eliminating false positives and resolving heterogeneity.

### Fluorescence imaging-guided photodynamic therapy based on HTP/CFN

**Figure 5:**
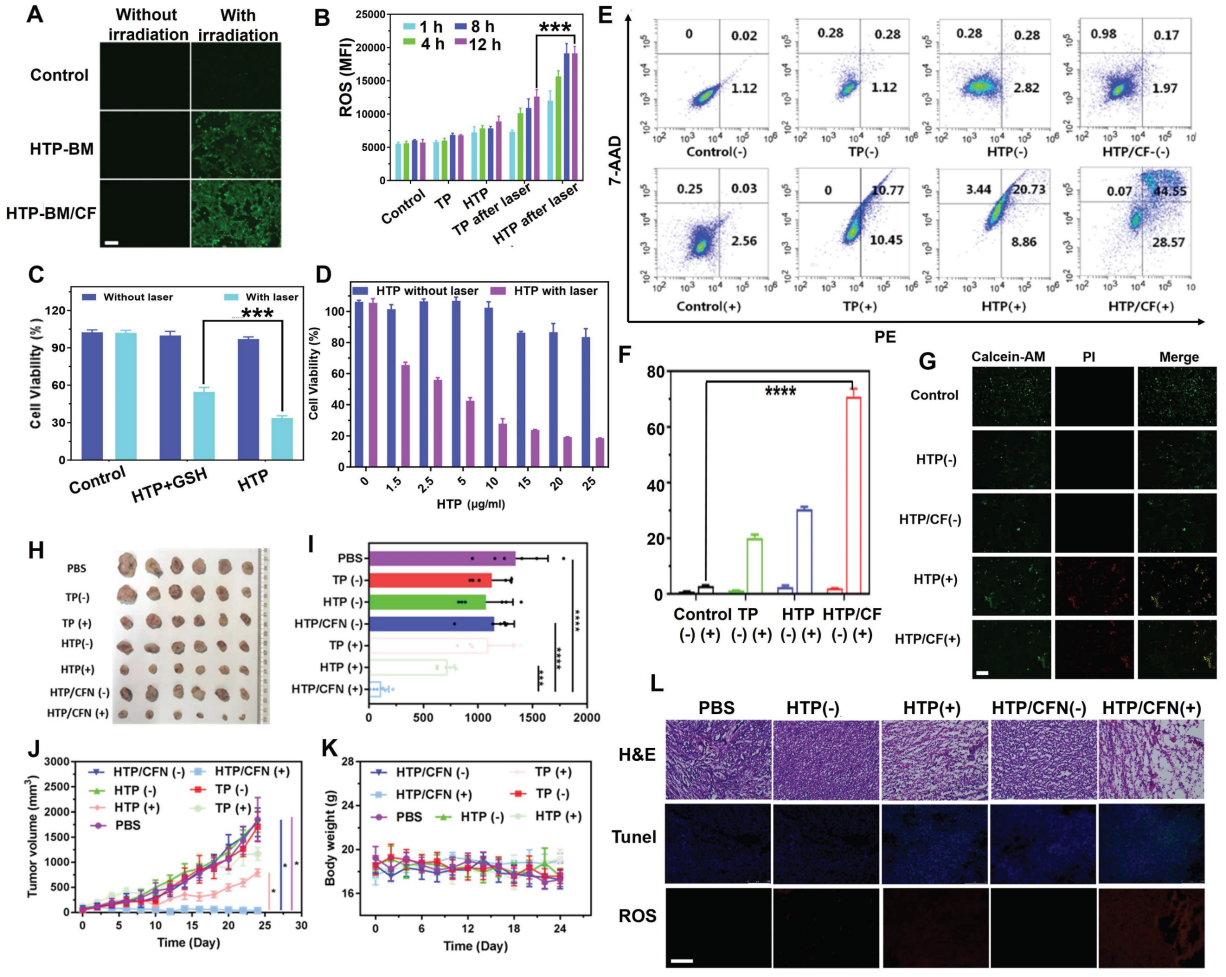
(A) Intracellular ROS fluorescence imaging after HTP-BM and HTP-BM/CF treatment for 12 h in the presence of laser exposure (660 nm, 1.2 W/cm^2^, 5 min), (scale bar: 500 µm). (B) Intracellular ROS fluorescence quantification by flow cytometry. (C) 4T1 cells viability incubated with HTP and GSH-treated HTP with or without laser irradiation (660 nm, 1.2 W/cm2, 8 min). (D) 4T1 cells cytotoxicity of HTP along the concentration with and without laser irradiation. (E) Apoptosis using FACS analysis of 4T1 cells treated with various formulations (PBS, TP, HTP, HTP/CF) before and after laser irradiation and (F) its quantitative fluorescence analysis. (G) Live (green) and dead (red) cells fluorescence images of 4T1 cells after incubation with HTP and HTP/CF with and without laser irradiation (660 nm, 1.2 W/cm^2^), (scale bar: 20 μm). (H) Tumor tissues image of mice extracted after 21 days of treatment with various formulations. (I) Tumor weight statistics of each group after 21 days of treatment. (J) Curves of tumor volume after different treatment with various formulations. (K) Changes in body weight of mice after treatment with different formulations (scale bar:100 µm). (L) Histological and immunohistochemical analysis of tumor sections collected from different treatment groups (scale bar:100 µm). Where (+) represents laser irradiation and (-) represents without laser irradiation. (* *p*<0.05, *** *p*<0.001, and **** *p*<0.0001)

Besides precise tumor surgical resection, another application of fluorescence imaging-guided PDT was evaluated in reactive oxygen species (ROS) generation during the photodynamic oxygen depletion process to kill tumor cells. We employed a green fluorescence ROS probe, DCFH-DA, to estimate ROS generated by HTP and HTP-BM/CF in 4T1 cells. As displayed in Figure 5A, in the absence of laser irradiation, 4T1 cells treated with HTP and assembled HTP-BM/CF produced a negligible fluorescence signal, suggesting little or no ROS production. In contrast green fluorescence was observed with laser irradiation in 4T1 cells treated with HTP and HTP-BM/CF with HTP-BM/CF-treated cells showing stronger fluorescence than the cells treated with HTP probably due to higher ROS generation. The reason for the increased fluorescence could be the improved solubility of HTP-BM/CF due to the hydrophilic CD-HPG shell and increased endocytosis by tumor cells, which was evident in the Figure 3G.

In addition, fluorescence intensities attributed to ROS generation were quantified by flow cytometry. Quantitative experiments presented in Figure 5B confirm that intracellular ROS production occurred in a time-dependent manner. The relative fluorescence intensity in the HTP-BM/CF cells after 12 h of laser irradiation was almost 6 times higher than those without irradiation and 1.5 times higher than the HTP-treated cells. These results confirmed that the HTP-BM/CF assemblies were highly potent and efficient in tumorolytic ROS induction.

Cytotoxicity experiment was also performed to assess the photodynamic killing effects of HTP on 4T1 tumor cells. As shown in Figure 5C, polymerized HTP maintained the photodynamic effects of the TP monomer and exhibited concentration-dependent killing efficiency under laser irradiation, decreasing cell viability to 18% at only 25 μg/mL. No significant cytotoxicity was observed in control cells in the absence of laser irradiation. These findings suggested that HTP-BM/CF can be used as a theranostic platform for fluorescence-guided photodynamic therapy.

Our previous study found that disulfide bonds could effectively sensitize the killing effects of chemotherapeutic drugs on cancer cells by depleting intracellular GSH 50. Herein, we further explored the impact of disulfide bonds on photodynamic therapy and found that the photodynamic killing effects of HTP pretreated with additional GSH (10 μM) significantly reduced tumor cell killing effects (Figure 5D). We speculated that disulfide bonds likely sensitize photodynamic therapy by perturbing redox homeostasis in tumor cells through GSH depletion.

Subsequently, apoptosis of 4T1 tumors treated with various formulations (PBS, TP, HTP, HTP/CF) in the absence and presence of laser irradiation was assessed by flow cytometry (Annexin V-7AAD/PE Apoptosis Detection Kit). Following 660 nm laser irradiation, the TP, HTP, HTP/CF-treated tumor cells underwent significant apoptosis (Figure 5E) due to phototoxic effects produced by the TP monomer photosensitizer. In contrast, the apoptosis rate in cells without laser irradiation appeared negligible. The highest apoptosis rate was observed in cells incubated with HTP-BM/CF in the presence of laser irradiation, with an apoptosis rate of 79.3 %, which was higher than the HTP (29.6 %) or TP-treated cells (21.2 %) (Figure 5F). The elevated apoptosis rate of HTP-BM/CF compared with other treatments with equivalent TP could be ascribed to the synergetic sensitization enabled by abundant disulfide linkage-caused GSH depletion and folate-mediated internalization enhancement. Thus, the apoptosis results analysis by flow cytometry further verified the cytotoxicity results.

We performed live/dead cell staining followed by fluorescence microscopy to assess the photodynamic treatment effects of HTP-BM/CF. As expected, green fluorescence was observed in the control, HTP, and HTP-BM/CF-treated cells without laser irradiation. In contrast, red fluorescence and less green signal were present in cells with HTP and HTP-BM/CF and laser irradiation (Figure 5G), with HTP-BM/CF exhibiting more red fluorescence than HTP. This enhanced phototherapeutic effect of HTP-BM/CF combined with the cytotoxicity and apoptosis results *in vitro* indicated their potential application for fluorescence imaging-guided photodynamic therapy.

Based on the *in vitro* PDT results, we further evaluated the anti-tumor activity of HTP-BM/CFN in 4T1 tumor-bearing mice with fluorescence imaging guidance (Figure 5H-K). An approximately 10-fold increase in tumor volume was observed in mice treated with PBS, TP (-), TP (+), HTP (-), and HTP-BM/CFN (-), suggesting that the therapeutic effects of TP alone were negligible regardless of light irradiation due to the poor solubility and low molecular weight of TP, prone to elimination during circulation. Comparatively, tumor growth in the HTP-mice under laser irradiation exhibited moderate inhibition, and eventually reached a size of ~468 mm^3^ which was less than half of the PBS group. Limited tumor inhibition by HTP was induced by poor tumor accumulation due to its non-specific interactions in the blood and restricted enhanced permeability and retention (EPR)-mediated tumor targeting. In contrast, mice treated with fluorescence imaging-guided photodynamic therapy with HTP-BM/CFN showed the most effective tumor suppression compared to other groups. This effect was mainly attributable to the two-stage AND logic gate design, consisting of active/passive dual tumor-targeting to maximize HTP-BM/CFN accumulation in tumors, and spatially tumor-specific imaging guidance and PDT efficiency feedback via fluorescence intensity variation, synergistically contributing to precise and monitorable phototheranostic treatment.

Representative hematoxylin and eosin (H&E) staining images of tumor sections showed extensive apoptosis/necrosis in HTP-BM/CFN-treated mice than in other groups, confirming the excellent anti-tumor efficacy mediated by HTP-BM/CFN. Moreover, ROS production inside tumor tissues was detected with a fluorescence probe after treatment with various formulations. Red fluorescence was visible by both HTP and HTP-BM/CFN treatments with laser irradiation. However, the fluorescence signal was significant with HTP-BM/CFN treatment under laser irradiation, indicating efficient ROS production within tumor tissues (Figure 5L).

TUNEL staining also showed a higher green fluorescence (cell apoptosis) intensity and more efficient apoptosis induction with HTP-BM/CFN than with other treatments. Thus, H&E, ROS, and TUNEL results indicated prominent *in vitro* and *in vivo* anti-tumor HTP-BM/CFN effects mediated by ROS-induced apoptosis, enabling by tumor-targeted enrichment and tumor-specific fluorescence imaging guidance.

### *In vivo* biocompatibility evaluation of HTP-BM/CFN

**Figure 6:**
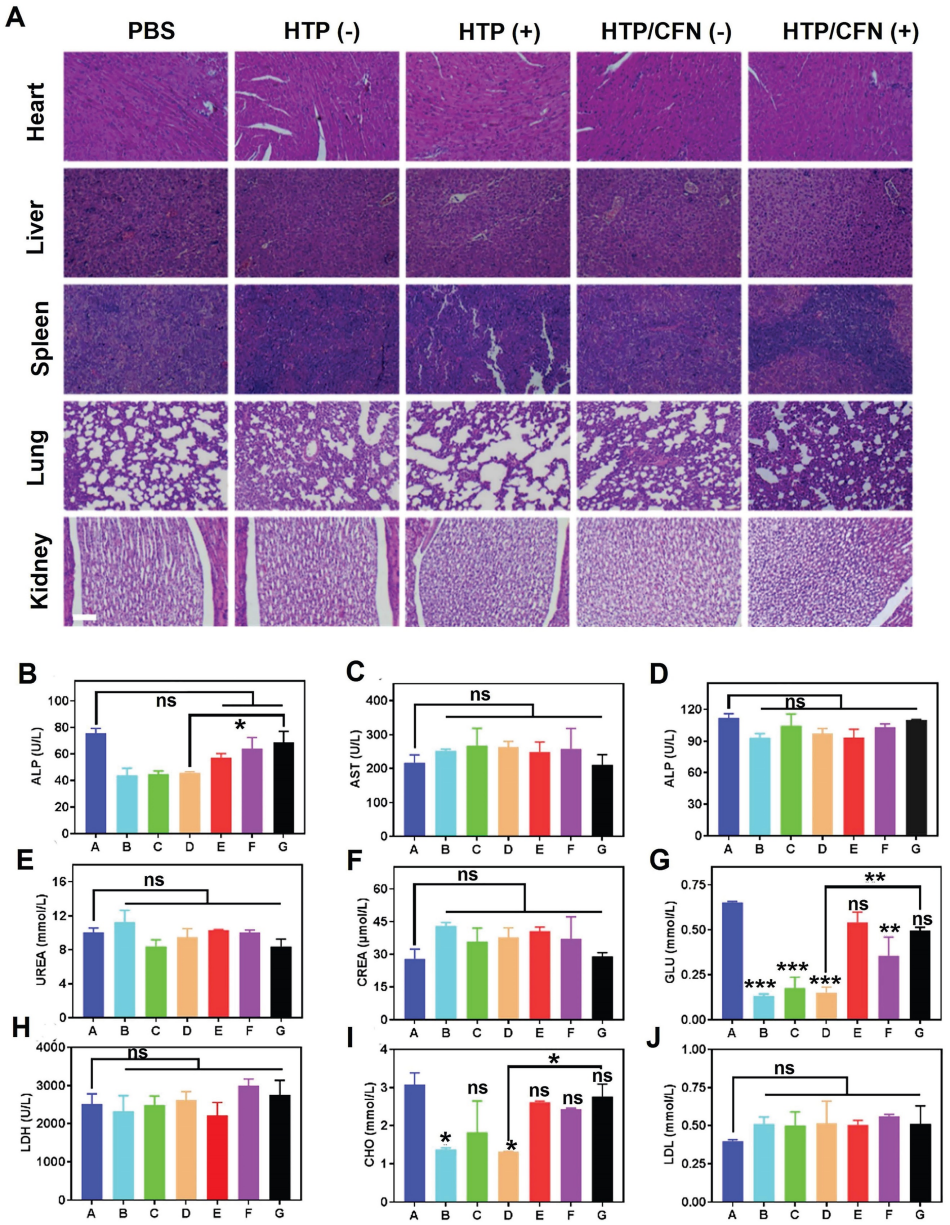
*In vivo* biocompatibility evaluation of the HTP-BM/CFN: (A) H&E staining images of major organs (heart, liver, spleen, lung, kidney) of mice in each treatment groups, where (+) represents light and (-) represents no light (scale bar is 100 µm) (B)-(J) Blood biochemical analysis of hepatic function (ALT, AST and ALP), renal function (UREA, CREA, and GLU), low-density lipoprotein (LDH), total cholesterol (CHO) and blood lipids (LDL) collected from mice treated with different formulations (A: saline group, B: TP(-) group, C: TP(+) group, D: HTP(-) group, E: HTP(+) group, F: HTP-BM/CFN (-) group and G: HTP-BM/CFN ( +) group) . (* *p*<0.05, ** *p*<0.01, *** *p*<0.001 and *ns p*>0.05)

We assessed the potential toxicity of various formulations (TP, HTP, HTP-BM/CFN) by monitoring the body weight of mice. No significant weight loss or other abnormalities were noticed in all groups during treatment (Figure 5K). Further, H&E staining of major organ sections (i.e. heart, liver, spleen, lung, and kidney) did not reveal any pathological abnormalities or lesions (Figure 6A), indicating negligible toxicity of HTP-BM/CFN. We collected blood samples from mice 24 h after intravenous injection and performed routine analyses. Functional indices of liver and kidney, deemed the most important metabolic organs, were within the normal range following HTP and HTP-BM/CFN treatment and did not differ statistically from the blank control (Figure 6B-J). These results indicated that HTP-BM/CFN had no significant side effects on liver and kidney of mice, suggesting no major toxicity concerns *in vivo*.

## Conclusion

We have successfully developed a modularly assembled theranostic agent (HTP-BM/CFN) for hypoxia fluorescence imaging-guided surgical resection and PDT against heterogeneous tumors. HTP-BM/CFN operated on a two-stage “AND” logic gate principle for improving tumor-specific fluorescence visualization accuracy and enabling tunability of fluorescence signal compensation and homogenization within tumors. There was compelling evidence from* in vitro* and *in vivo* analyses that the HTP-BM/CFN system endowed with biocompatibility and dual tumor targeting ability, could facilitate fluorescence turn-on specifically, signal compensation selectively, and extended the imaging time window needed for precise normalized image-guided treatment. HTP-BM/CFN also achieved enhanced PDT by depleting cellular GSH and elevating ROS production in tumor cells. We anticipate that this theranostic agent can achieve accurate and tunable tumor physiological signal imaging with theoretical implications for future research cancer diagnostics and treatment research, especially in the challenging context of tumor heterogeneity across disease stages.

## Materials and Methods

### Materials and Instruments

Tetra (4-aminophenyl) porphyrin and paraformaldehyde were purchased from Macklin Biochemical Co., Ltd (Shanghai, CN). DMEM, RPMI-1640 medium, and fetal bovine serum (FBS) were obtained from Thermo Fisher Scientific Inc. (U.S.A). Anhydrous ethanol, methanol, hydrochloric acid, and all other chemicals were purchased from the Chemical Reagent Factory of Guangzhou. Fluorescence labeling dyes Cy5.5 and FITC dye were acquired from Yuan Ye Bio-Technology Co. (Shanghai, CN). DCFH-DA was purchased from Sigma-Aldrich (St. Louis). Annexin V-PE/7-AAD Apoptosis Kit was obtained from KeyGen BioTech.

The synthetic products were analyzed by NMR spectrometer (Bruker-300 MHz, Germany), Fourier infrared spectrometer (VERTEX70, Bruker, Germany), and dynamic light scattering (Malvern, UK). The absorption spectrum and intensity were measured by a UV-vis spectrophotometer (UV-2550, Shimadzu, Japan). The morphology of assemblies was characterized by TEM (JEM-2010HT, JEOL, Japan). Fluorescence emission spectra of the samples before and after assembly were measured using a fluorescence spectrophotometer (F-7000, Hitachi, Japan). Gel permeation chromatography (Viscoteck GPC, Malvern, UK) was utilized to validate the dissociation of assemblies and HTP degradation. The tumor targeting delivery effect of HTP-BM/CFN was verified with an *in vivo* imaging system (IVIS Lumina III, PerkinElmer).

### Synthesis of HTP-BM and CFN

The HTP-BM was synthesized as illustrated in Scheme S1. N, N′-bis (acryloyl) cystamine (BAC) was synthesized as previously reported 51. Tetrakis (4-aminophenyl) porphyrin (TP) (13.6 mg) was dissolved in a mixture of 8 ml of methanol and 2 ml of dimethylamine (DMF). BAC (55 mg) was dissolved in methanol, and added dropwise to the TP solution under stirring for 24 h. Subsequently, the mixture was slowly added dropwise to ultrapure water followed by centrifugation (12000 rpm for 20 min) to collect polyporphyrin (HTP) precipitate. The crude products were washed and purified with acetone and dried under vacuum. Next, HTP (19.3 mg), carboxybenzimidazole (BM-COOH) (10 mg) and 1-(3-dimethylaminopropyl)-3-ethylcarbodiimide hydrochloride (EDC) (6.5 mg) were dissolved in 10 mL of dimethyl sulfoxide (DMSO) with stirring for 30 min. Then, N-hydroxysulfosuccinimide (NHS) (5 mg) was added to the above solution at room temperature for another 12 h. Subsequently, the mixture was slowly added into ultrapure water, centrifuged (12,000 rpm for 20 min), and washed three times with acetone to collect the product that was dried under vacuum.

Cyclodextrin-derived hyperbranched polyglyceryl ether (*β*-CD-HPG) was synthesized as shown in Scheme S2. In brief, 18-Crown-6 (1.0 g) and* β*-CD (0.5 g) were added to 25 mL of N, N-dimethylformamide (DMF). After the reaction mixture was heated to 90 ℃, 2 mL of DMF solution containing potassium hydride (0.168 g) was added, followed by intense stirring for 2 h under a nitrogen atmosphere. Next, 11 mL of glycidol was added by a micro syringe pump (1 mL/min) after lowering the temperature to 80 ℃, and the mixture was stirred for another 24 h. After the reaction was complete, ultrapure water was added until the mixture was completely dissolved and subjected to dialysis for 3 days (Mw = 1000 Da). The final product was collected through lyophilization.

The fluorescent probe CNP was synthesized in five steps as illustrated in Scheme S3 in the Supporting Information. The CNP and intermediate compounds were characterized using ^1^H NMR, ^13^C NMR, and high-resolution MS. The synthesis details and characterization results are presented in the Supporting Information (Figure S2-S13).

To obtain dual functionalized *β*-CD-HPG with a fluorescent probe and receptor modification (abbreviated as CFN) shown in Scheme S2, folic acid (10 mg), CNP (10 mg), and dicyclohexyl carbodiimide (DCC) (25 mg) were dissolved in 22 mL of dimethyl sulfoxide (DMSO) and stirred at room temperature for 30 min. Then, *β-*CD-HPG (140 mg) and 4-dimethylaminopyridine (DMAP) (14 mg) were added to the mixture and stirred at room temperature for another 24 h. Finally, the reaction mixture was purified by dialysis (Mw = 1000 Da) for 3 days before lyophilization.

### Preparation and characterization of shell-core HTP-BM/CFN

HTP-BM (0.05 mg) and *β-*CD-HPG-(FA/CNP) (0.5 mg) were dissolved in 0.5 mL of DMSO and stirred for 1h at room temperature. The obtained HTP-BM/CFN by self-assembly were dialyzed in 2000 Da dialysis tubes for 30 min and lyophilized for quantification. Two-dimensional nuclear Overhauser effect (NOE) spectroscopy nuclear magnetic resonance (2D-NOESY NMR, Bruker-300, Germany) was used to characterize the host-guest interaction between the* β-*CD and benzimidazole. Particle size and Zeta potential variation of host-guest assemblies with different assembly ratios were measured by DLS at room temperature. The morphology of optimized HTP-BN/CFN was observed by JEM-2010HT transmission electron microscopy (TEM). The HTP-BM/CFN assembly ratios used in the experiments below were set at the preferred 1:10 (*w/w*).

To confirm the NTR-responsive fluorescence turn-on, CNP, CFN, and HTP-BM/CFN solutions at a fixed concentration of 1.5 mg/mL were configured, and nitroreductase (NTR) and nicotinamide adenine dinucleotide (NADH) were added sequentially. The fluorescence emission spectra before and after self-assembly were measured using a fluorescence spectrophotometer (CNP detection parameters: Ex: 436 nm, detection range: 500-700 nm, Ex/Em slit: 5 nm, scan speed: 12000 nm/min).

### Photodynamic effect induced oxygen consumption analysis *in vitro*

The photodynamic effect of the assembled HTP-BM/CF was verified by detecting the oxygen consumption in an aqueous solution under laser irradiation. PBS with or without HTP-BM/CF (10 μM) was prepared in 15 mL centrifuge tubes with an oil seal on the liquid surface to prevent oxygen interference from the air. A 660 nm laser (1.2 W/cm^2^) was used to continuously irradiate the solutions, and the dissolved oxygen values were recorded every two minutes by a zero-calibrated JPB-607A portable dissolved oxygen meter. The experiment was terminated when no change in dissolved oxygen occurred in 5 min.

### Endocytosis and photodynamic effect of HTP-BM/CFN on 4T1 cells

4T1 cells were purchased from the Cell Resources Center of Shanghai Institute of Life Sciences (SIBS, China). The cells were cultured in RPMI-1640 medium containing 10 % fetal bovine serum and 1 % penicillin in a constant temperature incubator at 37 °C with 5 % CO_2_.

To study the uptake of the assemblies by 4T1 cells, Cy5.5-labelled HTP and HTP-BM/CFN assemblies dissolved in the complete medium were co-incubated with the cells and incubated for 0.5, 1, 2, and 4 h, and subsequently analyzed by flow cytometry. Cytotoxicity assay was performed using the CCK-8 assay kit to verify the toxicity of polyporphyrin in the presence or absence of laser irradiation. In addition, to verify the sensitizing effect of disulfide bonds in the polyporphyrin core on photodynamic processes, 4T1 cells were co-incubated with 20 μg/mL HTP, and one group was additionally treated with 10 μM GSH to analyze cell viability. For the apoptosis assay, the medium containing TP, HTP, and HTP/CF assemblies was co-incubated with 4T1 cells. Then the cells were incubated with the binding buffer containing 5 μL Annexin V/PE and 5 μL 7-AAD for 15 min, and finally apoptosis percentage was analyzed by flow cytometry. Cells were incubated with fresh culture medium containing HTP or HTP/CF (equivalent to 10 μM porphyrin), for 48 h, and then incubated with calcein-AM and PI for 15 min and 10 min, respectively. The live-dead cells were then observed by inverted fluorescence microscopy. Cells were treated with different materials for 2 h to measure intracellular ROS. The ROS probe DCFH-DA (10 μM) was added and incubated for 20 min, and after irradiation with 660 nm laser (1.2 W/cm^2^, 8 min), the fluorescence signals were observed by a fluorescence microscope.

### NTR activity in cells under photodynamic treatment

After photodynamic treatment, we compared the difference in NTR production in normal and tumor cells. 3T3 and 4T1 cells at a density of 1×10^6^ cells/well were inoculated in 12-well plates for 24 h. Subsequently, cells were incubated overnight with the fresh culture medium containing HTP and HTP/CF (equivalent to 10 μM porphyrin). After washing with PBS and replacing with fresh 1640 medium, cells were irradiated with a 660 nm laser (1.2 W/cm^2^) for 0, 5, 10, 15, 30, and 60 min. Subsequently, cells were collected by adding 500 μL of the nitroreductase extract, sonicated, centrifuged at 4 ℃ (2000 rpm, 5 min), and the supernatant was placed on ice. The mouse nitroreductase ELISA kit (ZOKEYO) was used, the nitrate reagent with NADH was added, and the absorbance at 450 nm was recorded using a microplate reader.

### Fluorescence turn-on and photodynamic-modulated signal enhancement of HTP-BM/CFN in 4T1 cells

We explored the fluorescence turn-on of HTP-BM/CFN in 4T1 cells under the hypoxic microenvironment, by placing the orifice plate in a sealed bag for 15 min in an anoxic gas-generating unit (purchased from Mitsubishi MGC, Japan). Then, the gas-generating part was removed, and the orifice plate in the sealed bag was incubated overnight. The HTP-BM/CFN containing complete medium was added to the 4T1 cells and incubated for 4 h. Subsequently, the cells were observed using an inverted fluorescence microscope.

To further investigate the influence of the photodynamic oxygen-consuming process on the fluorescence intensity after the uptake of fluorescent probe by tumor cells, 4T1 cells were seeded in a culture dish and incubated overnight. At different time points, CFN and HTP/CFN were introduced into the 4T1 cell culture medium and co-incubated for 4 h. The cells were washed three times with PBS and incubated with fresh culture medium for an additional 2 h. After capturing fluorescence images, the cells were subjected to 4 h quiescence. Subsequently, the two groups were irradiated with a 660 nm laser (1.2 W/cm^2^) for 8 min, followed by a 30-min interval. Then, the cells were incubated with DAPI for 15 min, fixed with 4 % paraformaldehyde, and observed using an inverted fluorescence microscope.

### Tumor-specific targeting, fluorescence turn-on and signal enhancement of HTP-BM/CFN *in vivo*

BALB/C mice (4-6 weeks) were provided by Beijing Huafukang Laboratory Animal Technology Co. Clinically relevant abnormalities in the animals were recorded daily or every other day during the study. Mice with tumor overgrowth (>2000 mm^3^) and weight loss of more than 20 % were euthanized. Tumor-bearing BALB/C mice were established by subcutaneously injecting, 1×10^6^ cells/mL 4T1 cell suspension in PBS into lateral hind limb buttocks of healthy mice.

The tumor-targeting of HTP-BM/CFN was verified using in an *in vivo* imaging system. Female mice bearing 4T1 tumors were intravenously injected with 2.5 mg/kg of Cy5.5-labelled HTP or Cy5.5-labelled HTP-BM/CFN. The fluorescence signal was tracked at various time points using an excitation wavelength of 685 nm, and fluorescence images were acquired up to 24 hours. Subsequently, the mice were euthanized, and their main organs and tumors were collected and imaged.

To confirm the tumor-specific fluorescence imaging, 200 μL of FITC (10 μM) and HTP-BM/CFN (10 μM) were injected simultaneously at the tumor site (left) and subcutaneous muscle tissue (right) of mice (n=3), When the tumor volume reached 200 mm^3^, an *in vivo* imaging system was used to acquire the fluorescence imaging and signals (Ex = 430 nm, Em = 520 nm) quantified at 0 h and 6 h after the injection. Mice were executed at 6 h after the injection of HTP-BM/CFN, and main organs and tumors were excised and imaged.

The Light-modulated fluorescence enhancement based on HTP-BM/CFN was verified* in vivo*. HTP-BM/CFN (10 μM), HTP-BM/CFN (10 μM) with light irradiation and FITC (10 μM) with light irradiation were injected into tumor-bearing mice (n=3). Fluorescence signals (Ex = 440 nm, Em = 520 nm) of HTP-BM/CFN and FITC were obtained using an *in vivo* imaging system at 0, 2 and 6 h after injection. Subsequently, 6 h after injection of the HTP-BM/CFN and FITC, the tumor site was irradiated with a 660 nm laser with an irradiation power of 1.2 W/cm^2^ for 10 min, and the fluorescence imaging was performed again at intervals of 2 h and 6 h after the laser irradiation (8h and 12h after injection).

The compensation effect of the light-modulated fluorescence enhancement technique on heterogeneous tumor imaging was assessed. Specifically, three mice exhibiting heterogeneous fluorescence signals were randomly selected after injecting 100 μL HTP-BM/CFN via the tail vein, and the fluorescence signals of HTP-BM/CFN (Ex = 440 nm, Em = 520 nm) were visualized using an *in vivo* imaging system at 6 h after injection, and the tumor site was irradiated with a 660 nm laser at the power of 1.2 W/cm^2^, and then the fluorescence imaging was performed at an interval of 1 h after the irradiation.

### Fluorescence imaging-guided tumor surgical resection

After the tumor volume reached 200 mm^3^, three tumor-bearing BALB/c mice were injected with HTP-BM/CFN (10 μM) via the tail vein, and subsequently fluorescence imaging was obtained using an *in vivo* imaging system. The tumor site was irradiated with a 660 nm laser for 10 min at 1.2 W/cm^2^ to locate the tumor tissue boundary. Fluorescence imaging was then performed at 1 h intervals after irradiation to assist in the surgical resection of the tumor.

### Fluorescence imaging-guided photodynamic therapy* in vivo*

After the tumor volume reached 200 mm^3^, mice were randomly divided into seven treatment groups (n = 6 per group) and injected with following formulations: (1) 200 μL of PBS; (2) 200 μL of pure TP (20 mg/kg); (3) 200 μL of pure TP (20 mg/kg) + laser irradiation; (4) 200 μL of HTP (20 mg/kg); (5) 200 μL of HTP (20 mg/kg) + laser irradiation; (6) 200 μL of HTP-BM/CFN (20 mg/kg); (7) 200 μL of HTP-BM/CFN (20 mg/kg) + laser irradiation. The mice in the laser groups were irradiated with 660 nm laser at the tumor site six hours after injection under fluorescence imaging guidance. The irradiation power was 1.2 W/cm^2^, and the irradiation time was 10 minutes, performed once every 3 days for a total of 2 times. The tumor volume and body weight of the mice were recorded every 2 days. On the 25th day, the mice were sacrificed, and the tumors were removed for H&E and immunofluorescence (ROS and Tunnel) analysis to assess the treatment effect in each group.

### Biocompatibility evaluation

For histological analysis, the major organs of mice, including the heart, liver, spleen, lung, and kidney, were collected and analyzed by H&E tissue staining. For evaluating the biochemical indicators, 300 μL of blood was collected from mice by eyeball blood sampling, and serum was separated by centrifugation at 2000 g for 15 min and stored at -80 ℃. The samples were tested by biochemical analyzer for liver function indicators, including glutamic oxaloacetic transaminase (ALT), glutamic alanine transaminase (AST), and alkaline phosphatase (ALP), kidney function indicators, including urea nitrogen (BUN), uric acid (UREA), and creatinine (Cr), lipid profile, including (LDL) low-density lipoprotein, (HDL) high-density lipoprotein, total cholesterol (CHO).

### Statistical analysis

Each of the above experiments was repeated at least three times (n ≥ 3) and all data are presented as mean ± standard deviation (SD). Statistical analyses were processed through GraphPad 7.0 using One-way ANOVA to compare different experimental conditions. In each statistical analysis, statistical significance was defined as * *p*<0.05, ** *p*<0.01, *** *p*<0.001, **** *p*<0.0001 and *ns* p>0.05.

## Supplementary Material

Supplementary methods and figures.

## Abbreviations

BAC: N, N'-bis(acryloyl) cystamine
TP: 5,10,15,20-tetra (4-aminophenyl) porphyrin
BM: Benzimidazole
FA: Folic acid
HTP: Disulfide-bonded polyporphyrin
HTP-BM: Benzimidazole group modified HTP
*β*-CD-HPG: *β*-cyclodextrin-derived hyperbranched glycerol ether
NTR: Nitroreductase
CNP: NTR-activatable fluorescent probe
CF: *β*-CD-HPG modified with folic acid
CFN: *β*-CD-HPG modified with folic acid and fluorescent probe
HTP-BM/CFN: Core-shell assemblies with HTP-BM and CFN
NADH: Nicotinamide adenine dinucleotide

## References

[1] Pe'er D, Ogawa S, Elhanani O, Keren L, Oliver TG, Wedge D (2021). Tumor heterogeneity. Cancer cell.

[2] Arrieta VA, Dmello C, McGrail DJ, Brat DJ, Lee-Chang C, Heimberger AB (2023). Immune checkpoint blockade in glioblastoma: from tumor heterogeneity to personalized treatment. The Journal of Clinical Investigation.

[3] Guo L, Kong D, Liu J, Zhan L, Luo L, Zheng W (2023). Breast cancer heterogeneity and its implication in personalized precision therapy. Experimental hematology & oncology.

[4] Ramón y Cajal S, Sesé M, Capdevila C, Aasen T, De Mattos-Arruda L, Diaz-Cano SJ (2020). Clinical implications of intratumor heterogeneity: challenges and opportunities. Journal of Molecular Medicine.

[5] Marusyk A, Janiszewska M, Polyak K (2020). Intratumor heterogeneity: the rosetta stone of therapy resistance. Cancer cell.

[6] Nicholson JG, Fine HA (2021). Diffuse glioma heterogeneity and its therapeutic implications. Cancer discovery.

[7] Carter B, Zhao K (2021). The epigenetic basis of cellular heterogeneity. Nature Reviews Genetics.

[8] Liang Y, Zhang H, Song X, Yang Q (2020). Metastatic heterogeneity of breast cancer: Molecular mechanism and potential therapeutic targets. Seminars in cancer biology: Elsevier.

[9] Tao J, Yang G, Zhou W, Qiu J, Chen G, Luo W (2021). Targeting hypoxic tumor microenvironment in pancreatic cancer. Journal of hematology & oncology.

[10] Zou M-Z, Liu W-L, Chen H-S, Bai X-F, Gao F, Ye J-J (2021). Advances in nanomaterials for treatment of hypoxic tumor. National Science Review.

[11] Roy S, Kumaravel S, Sharma A, Duran CL, Bayless KJ, Chakraborty S (2020). Hypoxic tumor microenvironment: implications for cancer therapy. Experimental Biology and Medicine.

[12] Cheng Y, Kong X, Chang Y, Feng Y, Zheng R, Wu X (2020). Spatiotemporally synchronous oxygen self-supply and reactive oxygen species production on Z-scheme heterostructures for hypoxic tumor therapy. Advanced Materials.

[13] Hompland T, Fjeldbo CS, Lyng H (2021). Tumor hypoxia as a barrier in cancer therapy: why levels matter. Cancers.

[14] Chen H, Guo Q, Chu Y, Li C, Zhang Y, Liu P (2022). Smart hypoxia-responsive transformable and charge-reversible nanoparticles for the deep penetration and tumor microenvironment modulation of pancreatic cancer. Biomaterials.

[15] Ding Y, Yu W, Wang J, Ma Y, Wang C, Wang Y (2022). Intelligent supramolecular nanoprodrug based on anionic water-soluble [2] biphenyl-extended-pillar [6] arenes for combination therapy. ACS Macro Letters.

[16] Ding Y, Yu W, Shen R, Zheng X, Zheng H, Yao Y (2024). Hypoxia-Responsive Tetrameric Supramolecular Polypeptide Nanoprodrugs for Combination Therapy. Advanced Healthcare Materials.

[17] Zhou H, Guo M, Li J, Qin F, Wang Y, Liu T (2021). Hypoxia-triggered self-assembly of ultrasmall iron oxide nanoparticles to amplify the imaging signal of a tumor. Journal of the American Chemical Society.

[18] Chen S, Chen M, Yang J, Zeng X, Zhou Y, Yang S (2021). Design and engineering of hypoxia and acidic pH dual-stimuli-responsive intelligent fluorescent nanoprobe for precise tumor imaging. Small.

[19] Wei D, Sun Y, Zhu H, Fu Q (2023). Stimuli-Responsive Polymer-Based Nanosystems for Cancer Theranostics. ACS nano.

[20] Yang Y, Wu H, Liu B, Liu Z (2021). Tumor microenvironment-responsive dynamic inorganic nanoassemblies for cancer imaging and treatment. Advanced Drug Delivery Reviews.

[21] Zhu H, Li Q, Shi B, Ge F, Liu Y, Mao Z (2020). Dual-emissive platinum (II) metallacage with a sensitive oxygen response for imaging of hypoxia and imaging-guided chemotherapy. Angewandte Chemie International Edition.

[22] Jin Y, Li D, Zheng X, Gao M, Wang W, Zhang X (2024). A Novel Activatable Nanoradiosensitizer for Second Near-Infrared Fluorescence Imaging-Guided Safe-Dose Synergetic Chemo-Radiotherapy of Rheumatoid Arthritis. Advanced Science.

[23] Zou J, Li Z, Zhu Y, Tao Y, You Q, Cao F (2024). pH/GSH dual responsive nanosystem for nitric oxide generation enhanced type I photodynamic therapy. Bioactive Materials.

[24] Morsby JJ, Zhang Z, Burchett A, Datta M, Smith BD (2024). Ratiometric near-infrared fluorescent probe for nitroreductase activity enables 3D imaging of hypoxic cells within intact tumor spheroids. Chemical Science.

[25] Li X, Huo F, Zhang L, Yin C (2024). Hypoxia-activated and improved cell uptake near-infrared fluorescent probe for precise guiding surgical resection. Nano Today.

[26] Zhong D, Li W, Qi Y, He J, Zhou M (2020). Photosynthetic biohybrid nanoswimmers system to alleviate tumor hypoxia for FL/PA/MR imaging-guided enhanced radio-photodynamic synergetic therapy. Advanced Functional Materials.

[27] Xie H, Li Q, Yang H, Gao W, Zhang Q, Zhang P (2023). Mitochondrial-targeting fluorescent probe awakened by nitroreductase for hypoxic imaging and surgical navigation of solid tumors. Sensors and Actuators B: Chemical.

[28] Dai Y, Leng D, Guo Z, Wang J, Gu Y, Peng Y (2024). NIR-II excitation self-assembly nanomedicine for targeted NIR-IIa fluorescence imaging-guided cuproptosis-promoted synergistic therapy against triple-negative breast cancer. Chemical Engineering Journal.

[29] Ma T, Xia T (2021). Nanoparticle-Based Activatable Probes for Bioimaging. Advanced biology.

[30] Yin Y, Hu B, Yuan X, Cai L, Gao H, Yang Q (2020). Nanogel: A versatile nano-delivery system for biomedical applications. Pharmaceutics.

[31] Li D, Zhang R, Liu G, Kang Y, Wu J (2020). Redox-responsive self-assembled nanoparticles for cancer therapy. Advanced healthcare materials.

[32] Ding Y, Pan Q, Gao W, Pu Y, Luo K, He B (2023). Reactive oxygen species-upregulating nanomedicines towards enhanced cancer therapy. Biomaterials Science.

[33] Wang W, Zhou S, Cheng Z, Ma D, Liu T (2023). A glutathione-sensitive cationic polymer delivery of CRISPR-Cas9 RNA plasmid for targeting nasopharyngeal carcinoma gene therapy. Colloids and Surfaces B: Biointerfaces.

[34] Deng Y, Song P, Chen X, Huang Y, Hong L, Jin Q (2020). 3-Bromopyruvate-conjugated nanoplatform-induced pro-death autophagy for enhanced photodynamic therapy against hypoxic tumor. ACS nano.

[35] Gao D, Chen T, Chen S, Ren X, Han Y, Li Y (2021). Targeting hypoxic tumors with hybrid nanobullets for oxygen-independent synergistic photothermal and thermodynamic therapy. Nano-Micro Letters.

[36] Du J, Shi T, Long S, Chen P, Sun W, Fan J (2021). Enhanced photodynamic therapy for overcoming tumor hypoxia: From microenvironment regulation to photosensitizer innovation. Coordination Chemistry Reviews.

[37] Zou J, Zhu J, Yang Z, Li L, Fan W, He L (2020). A phototheranostic strategy to continuously deliver singlet oxygen in the dark and hypoxic tumor microenvironment. Angewandte Chemie International Edition.

[38] Huang L, Zhao S, Wu J, Yu L, Singh N, Yang K (2021). Photodynamic therapy for hypoxic tumors: Advances and perspectives. Coordination Chemistry Reviews.

[39] Lu J, Miao Y, Li Y Cuproptosis: Advances in Stimulus-Responsive Nanomaterials for Cancer Therapy. Advanced Healthcare Materials. 2024: e2400652.

[40] Liang J, Wu C, Zhou X, Shi Y, Xu J, Cai X (2021). Host-Guest Interaction-Based Dual response core/shell nanoparticles as efficient siRNA carrier for killing breast cancer cells. Colloids and Surfaces B: Biointerfaces.

[41] Guo Y, Cao X, Zheng X, Abbas SJ, Li J, Tan W (2022). Construction of nanocarriers based on nucleic acids and their applications in nanobiology delivery systems. National Science Review.

[42] Pang L, Tang X, Yao L, Zhou L, Hu S, Zhao S (2023). Smart down/upconversion nanomachines integrated with “AND” logic computation and enzyme-free amplification for NIR-II fluorescence-assisted precise and enhanced photodynamic therapy. Chemical Science.

[43] Chen J, Fu S, Zhang C, Liu H, Su X (2022). DNA logic circuits for cancer theranostics. Small.

[44] Hung M-C, Mills GB, Yu D (2009). Oxygen sensor boosts growth factor signaling. Nature medicine.

[45] Zheng J, Shen Y, Xu Z, Yuan Z, He Y, Wei C (2018). Near-infrared off-on fluorescence probe activated by NTR for *in vivo* hypoxia imaging. Biosensors and Bioelectronics.

[46] Li Z, Seehawer M, Polyak K (2022). Untangling the web of intratumour heterogeneity. Nature cell biology.

[47] Lan J-S, Liu L, Zeng R-F, Qin Y-H, Hou J-W, Xie S-S (2021). Tumor-specific carrier-free nanodrugs with GSH depletion and enhanced ROS generation for endogenous synergistic anti-tumor by a chemotherapy-photodynamic therapy. Chemical Engineering Journal.

[48] Li Y, He G, Fu L-H, Younis MR, He T, Chen Y (2022). A microneedle patch with self-oxygenation and glutathione depletion for repeatable photodynamic therapy. ACS nano.

[49] Zhu J, Xiao T, Zhang J, Che H, Shi Y, Shi X (2020). Surface-charge-switchable nanoclusters for magnetic resonance imaging-guided and glutathione depletion-enhanced photodynamic therapy. ACS nano.

[50] Xu Z, Zhang Y, Hu Q, Tang Q, Xu J, Wu J (2017). Biocompatible hyperbranched polyglycerol modified β-cyclodextrin derivatives for docetaxel delivery. Materials Science and Engineering: C.

[51] Wang W, Cai J, Wong N-K, Hong M, Deng J, Jin L (2022). Visualizing nitroreductase activity in living cells and tissues under hypoxia and hepatic inflammation. Analyst.

