# Supplementary material for: Modularized supramolecular assemblies for hypoxia-activatable fluorescent visualization and image-guided theranostics
Source: Theranostics. 2024 Jun 11;14(9):3634–52. doi: 10.7150/thno.95590 (PMC11209709; doi:10.7150/thno.95590)
Supplement: Supplementary file 1 — Supplementary methods and figures. [file thnov14p3634s1.pdf]

Supplementary Information

Modularized supramolecular assemblies for hypoxia-activatable fluorescent visualization and image-guided theranostics

Wen Liu [a,1], Bincheng Wang [a,1], Bei Guo [a,1], Junbin Zhu [b, e], Zejun Xu [c, f], Jiayue Xu [a], Zhen Wang [a], Guodong Sun [b, e], Wei Wang [d] *, Yi Zhang [a, b] *, Wei Xue [a] *

[a] Engineering Technology Research Center of Drug Carrier of Guangdong, Department of Biomedical Engineering, Jinan University, Guangzhou 510632, China

[b] China Guangdong Provincial Key Laboratory of Spine and Spinal Cord Reconstruction, The Fifth Affiliated Hospital (Heyuan Shenhe People's Hospital), Jinan University, Heyuan 517000, China

[c] College of Pharmacy, Jinan University, Guangzhou 510630, China

[d] Guangdong Provincial Key Laboratory of Optical Fiber Sensing and Communication, Institute of Photonics Technology, Jinan University, Guangzhou 510632, China

[e] Department of Orthopedics, The First Affiliated Hospital, Jinan University, Guangzhou 510630, China

[f] Bai Yun Shan Pharmaceutical General Factory, Guangzhou Bai Yun Shan Pharmaceutical Holdings Co. Ltd. Guangzhou 510515, China

Corresponding authors:

Wei Wang (wangwei_0415@163.com)

Yi Zhang (zhangyi_0424hot@163.com) ORCID: 0000-0003-4769-6642

Wei Xue (weixue_jnu@aliyun.com)

[1] These authors contributed equally to this work and should be considered co-first authors.

Supplementary Experimental Section

Synthesis of NTR fluorescent probe (CNP)

4-Bromo-1,8-naphthalic anhydride (2.77 g, 10.0 mmol) and tert-butyl 4-aminobutyrate (2.36 g, 14.8 mmol) were added to 150 mL of ethanol and refluxed under stirring for 4 h. The concentrated residue was purified by silica gel column chromatography to give a white solid (3.13 g, yield = 74.83). ^{1}H NMR (600 MHz, $CDCl_3$) δ: 8.66 (d, *J* = 7.3 Hz, 1H), 8.56 (d, *J* = 8.5 Hz, 1H), 8.41 (d, *J* = 7.8 Hz, 1H), 8.04 (d, *J* = 7.9 Hz, 1H), 7.90–7.79 (m, 1H), 4.24 (t, 2H), 2.37 (t, *J* = 7.5 Hz, 2H), 2.13–1.93 (m, 2H), 1.44 (s, 9H); ^{13}C NMR (151 MHz, $CDCl_3$) δ: 172.13, 163.58, 163.55, 133.24, 132.05, 131.24, 131.08, 130.62, 130.23, 129.02, 128.05, 123.08, 122.22, 80.31, 39.79, 33.20, 28.07, 23.54; HRMS: *m/z* calcd for $C_{20}H_{20}BrNO_4^+$: 440.0576 $[M+Na]^+$, found: 440.0468.

Next, ethanolamine (688.6 mg, 11.3 mmol) amd the white compound from the previous step (1.71 g, 4.0 mmol) was added to 4 mL of 2-methoxyethanol, the mixture solution was stirred and refluxed under argon atmosphere for 12 h. After the reaction, the yellow solid was washed with water and filtered. The yellow organic was purified by silica gel column chromatography to obtain an orange solid (1.20 g, yield = 75.0%). ^{1}H NMR (600 MHz, DMSO-d_6) δ: 8.69 (d, *J* = 7.5 Hz, 1H), 8.43 (d, *J* = 6.2 Hz, 1H), 8.25 (d, *J* = 8.5 Hz, 1H), 7.77 (t, *J* = 5.6 Hz, 1H), 7.72–7.64 (m, 1H), 6.81 (d, *J* = 8.7 Hz, 1H), 4.94 (t, *J* = 5.6 Hz, 1H), 4.03 (t, *J* = 7.0 Hz, 2H), 3.70 (q, *J* = 5.9 Hz, 2H), 3.47 (q, *J* = 5.9 Hz, 2H), 2.25 (t, *J* = 7.3 Hz, 2H), 1.93–1.73 (m, 2H), 1.34 (s, 9H); ^{13}C NMR (151 MHz, DMSO-d_6) δ:176.95, 169.10, 168.23, 156.05, 139.46, 135.91, 134.72, 133.88, 129.45, 127.11, 125.35, 112.86, 109.10, 84.72, 64.01, 50.77, 43.81, 37.78, 32.90, 28.43; HRMS: *m/z* calcd for $C_{22}H_{26}N_2O_5^+$: 422.0576 $[M+Na]^+$, found: 421.1734.

The orange compound (1184.3 mg, 2.97 mmol) from the previous step, carbon tetrabromide (1477.4 mg, 4.46 mmol) and riphenylphosphine (1168.5 mg, 4.46 mmol) were dissolved in 7 mL of DMF, respectively, and mixed together with stirring at 0 °C under argon. The reaction was carried out at room temperature for 5.5 h. Then, the mixture soluton was diluted with 100 mL EA, and washed with water and brine, respectively, and concentrated under reduced pressure. The feedstock was purified by flash chromatography (DCM: EtOH = 40:1) to obtain a yellow solid (970.7 mg, yield = 70.7%). ^{1}H NMR (600 MHz, $CDCl_3$) δ: 8.59 (d, *J* = 7.3 Hz, 1H), 8.47 (d, *J* = 8.3 Hz, 1H), 8.16 (d, *J* = 8.4 Hz, 1H), 7.66 (t, *J* = 7.8 Hz, 1H), 6.75 (d, *J* = 8.4 Hz, 1H), 5.70 (s, 1H), 4.22 (t, *J* = 7.3 Hz,

2H), 3.89 (t, 2H), 3.76 (t, *J* = 6.0 Hz, 2H), 2.37 (t, *J* = 7.7 Hz, 2H), 2.11–1.97 (m, 2H), 1.44 (s, 9H); ^{13}C NMR (151 MHz, $CDCl_3$) δ: 172.42, 164.53, 163.99, 148.34, 134.16, 131.33, 129.76, 125.91, 125.17, 123.17, 120.53, 111.50, 104.54, 80.24, 44.65, 39.42, 33.35, 30.85, 28.08, 23.74; HRMS: *m/z* calcd for $C_{22}H_{25}BrN_2O_4^+$: 483.0576 $[M+Na]^+$, found: 483.0890.

The yellow solid (834.4 mg. 1.81 mmol) from the previous step was dissolved in N,N-dimethylformamide together with 2-nitroimidazole (308.7 mg, 2.73 mmol), sodium iodide (91.43 mg, 0.61 mmol) and potassium carbonate (500.32 mg, 3.62 mmol). The mixture was stirred at 60 °C for 48 h. After the reaction, the mixture diluted with 60 mL DCM, washed with water and saturated NaCl brine, respectively, and filtered. The residue was purified by flash chromatography to give the Boc group pertected product as yellow solid (yield = 70.0%). ^{1}H NMR (600 MHz,$CDCl_3$) δ:8.63 (d, *J* = 6.3 Hz, 1H), 8.52 (d, *J* = 8.2 Hz, 1H), 8.03 (d, *J* = 8.2 Hz, 1H), 7.68 (t, 1H), 7.16 (d, *J* = 1.1 Hz, 1H), 7.01 (d, *J* = 1.1 Hz, 1H), 6.81 (d, *J* = 8.3 Hz, 1H), 5.65 (t, *J* = 5.9 Hz, 1H), 4.86 (t, *J* = 5.7 Hz, 2H), 4.24 (t, *J* = 7.2 Hz, 2H), 3.75 (q, *J* = 7.0 Hz, 2H), 2.37 (t, *J* = 7.7 Hz, 2H), 2.09–2.00 (m, 2H), 1.45 (s, 9H); ^{13}C NMR (151 MHz, $CDCl_3$) δ:172.36, 164.40, 163.90, 147.72, 133.96, 131.52, 129.81, 129.00, 126.52, 125.61, 125.58, 123.37, 120.51, 112.23, 104.12, 80.24, 58.51, 48.11, 43.64, 39.47, 33.32, 28.09, 23.72; HRMS: *m/z* calcd for $C_{25}H_{27}N_5O_6^+$: 516.1961 $[M+Na]^+$, found: 516.1854.

The fluorescent probe protected by Boc group is hydrolyzed under acidic conditions, and the fluorescent probe CNP modified by the carboxylic acid base group is synthesized. Specifically, the yellow solid (35 mg) from the previous step was dissolved in DCM/TFA (1:1, v:v) solution, and stirred for 4 h at room temperature. After the reaction, the organic solvent is evaporated by the rotary evaporator under reduced pressure to obtain a yellow product (CNP). The product is used for the next reaction without further purified.

Stability of HTP-BM/CFN under different physiological conditions

It is important to test the stability of prepared nanoparticles under different physiological conditions, especially when considering their use in biomedical applications. We simulated in vivo serum conditions as well as tumor acidic conditions to assess the stability of the assemblies under different physiological conditions by measuring the changes in particle size. The specific operation was as follows: the samples were dissolved in RPMI-1640 medium containing 10 % fetal bovine serum and PBS with pH=6.5. The hydrodynamic sizes were measured at different time points (1, 12,

24, 36, and 48 h), respectively, as shown in the figure S14, there was no obvious change of the assemblies' particle sizes in serum solution within 48 h, which proved that the assemblies had a good stability, which is conducive to their long circulation in vivo, and provides an important reference for the effectiveness and safety of their application in organisms. However, the particle size of the assemblies changed under acidic physiological environment, which may be due to the fact that the assemblies were deconstructed under acidic environment, and the HTP in the core would be agglomerated, which caused the increase of the particle size.

HTP-BM/CF catabolism at normal pH and high GSH concentration

To assess the decomposition of the HTP-BM/CF under normal pH and high concentration GSH conditions, the HTP-BM/CF was incubated in the buffer solution with pH at 7.4 (normal pH) and high GSH concentration of 10 μM for 4 h . The HTP-BM/CF treated as below and the untreated HTP-BM/CF were both measured using gel permeation chromatography (GPC). As shown in the Figure S15 below, the untreated assembly at pH=7.4 had only one peak, which retention time were 8.7 min. The peaks of HTP-BM/CF treated under normal pH and high concentration GSH appeared to shifted right and divided to two peaks in comparison to untreated HTP-BM/CF, indicating the molecular weight reduction of HTP-BM/CF. The two peaks of treated HTP-BM/CF may be assigned to CF (left) and the TP (right), in which the right peak is the decomposition product of HTP-BM inner core that was degraded by the high concentration GSH to generate TP. Based on this GPC result, it proves that HTP-BM/CF occurs decomposition under normal pH and high concentration GSH.

Release of self-assembled nanoparticles over time.

As requested, we tested the TP release of self-assembled HTP-BM/CF over time under in vitro (pH 7.4 without GSH) and in vivo (tumor environment (pH=6.5 and 10 μM GSH)) simulated conditions. As can be seen in the figure S16, the release of TP from the nano-assemblies in the PBS group has been stably kept at a low state, while under the condition of acidity plus high concentration of GSH, the self-assembled nanoparticles will gradually release porphyrin over time, and then the release rate will gradually slow down at a later stage to form a stable release phase, which also indicates that the stability of the assemblies is regulated by PH and GSH.

Supplementary Scheme Section

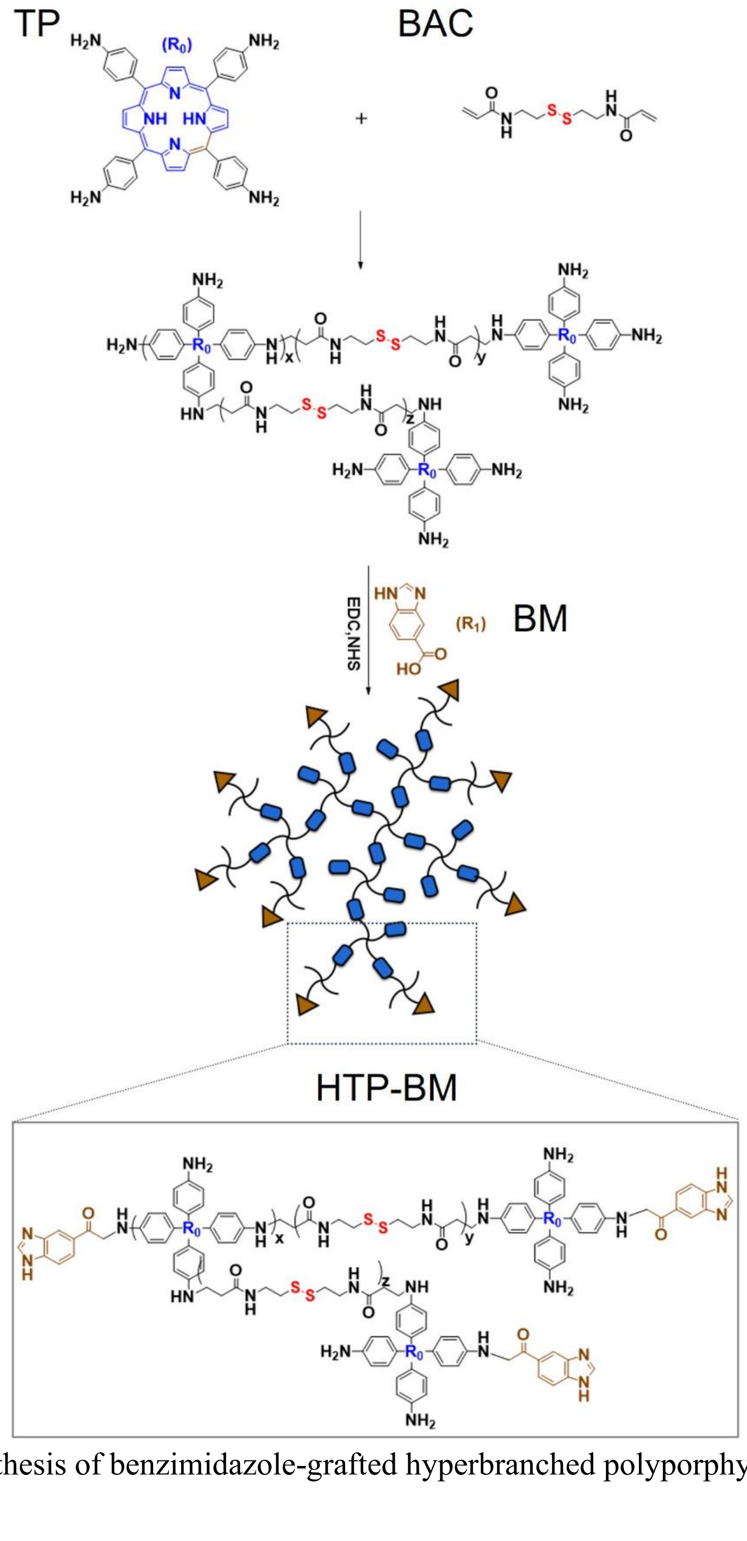

Scheme S1. Synthesis of benzimidazole-grafted hyperbranched polyporphyrins (HTP-BM).

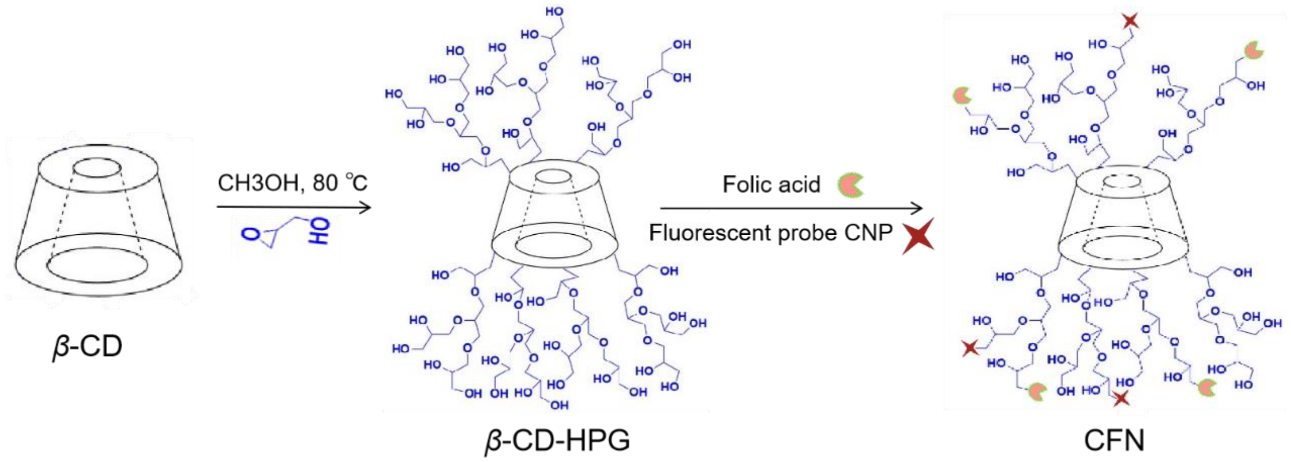

Scheme S2. Synthesis of targeted optical switch housing molecules.

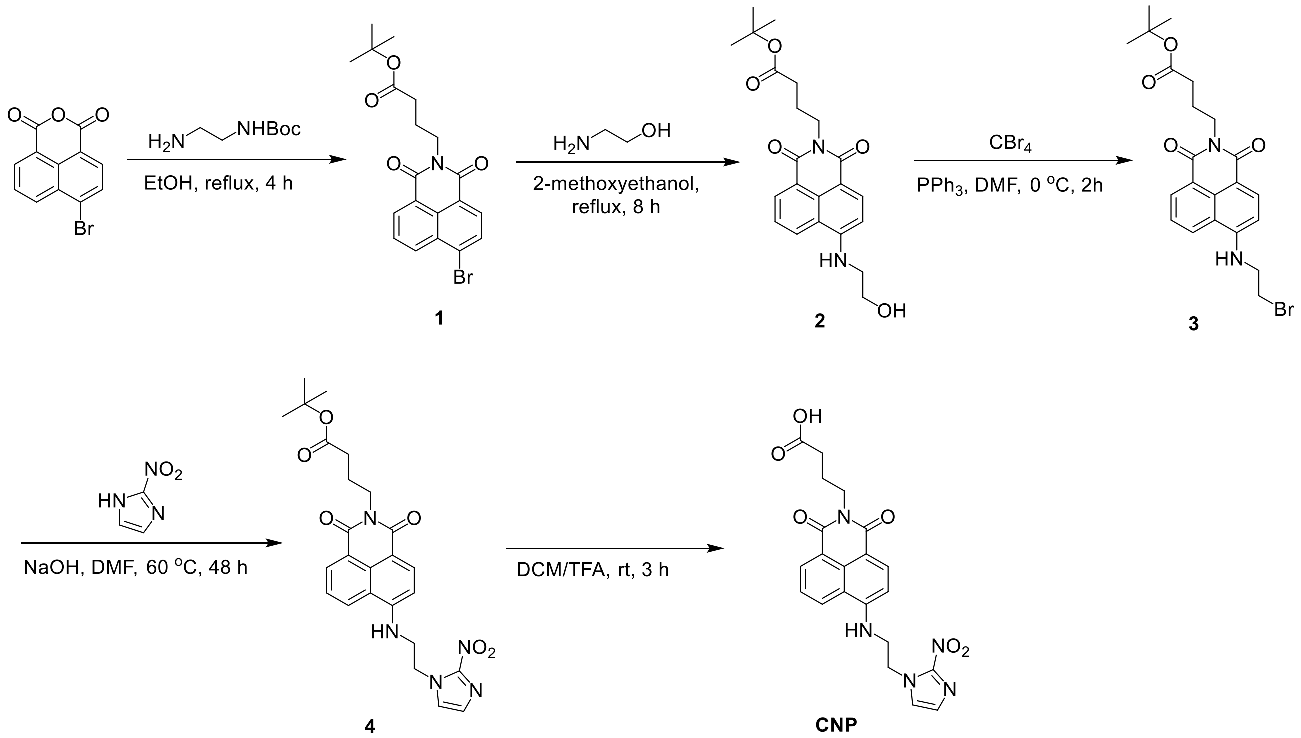

Scheme S3. Synthesis route of fluorescent probe CNP.

Supplementary Figure

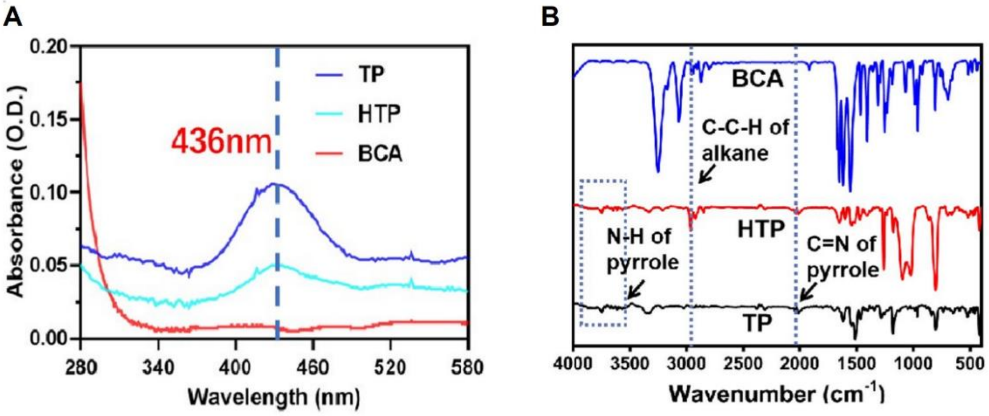

Figure S1. (A)UV and (B) infrared spectra of TP, BCA and HTP.

NMR spectra

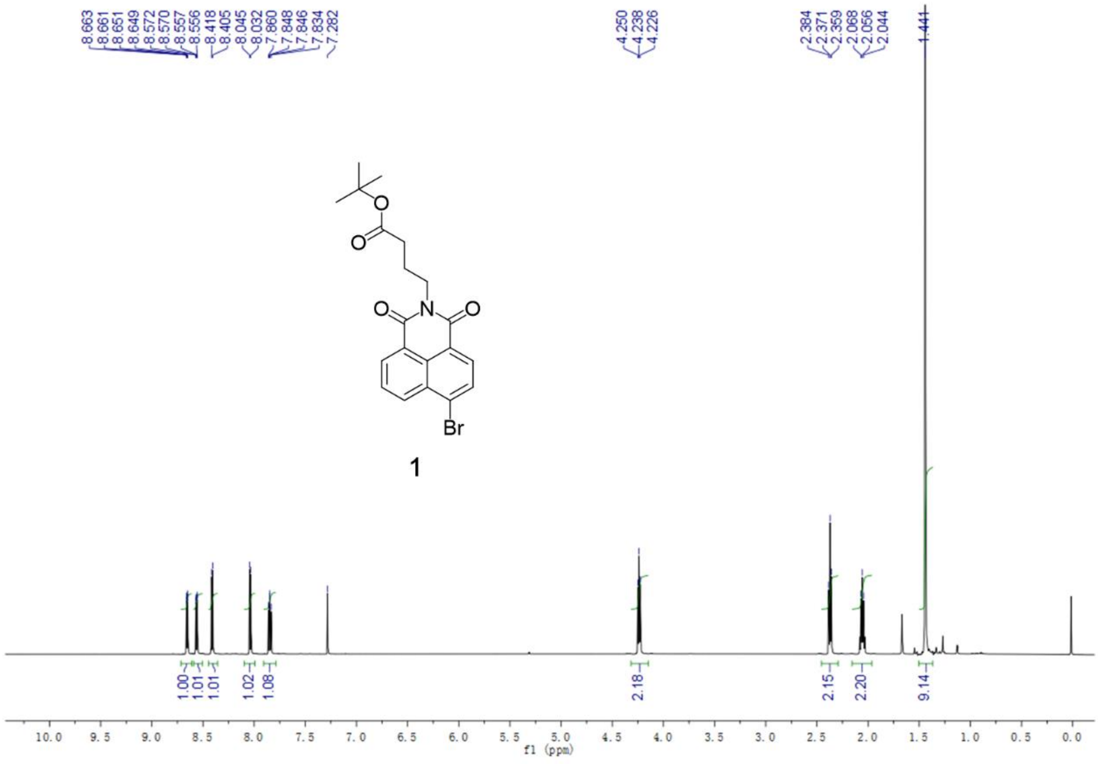

Figure S2. ^{1}H NMR spectra of compound **1**.

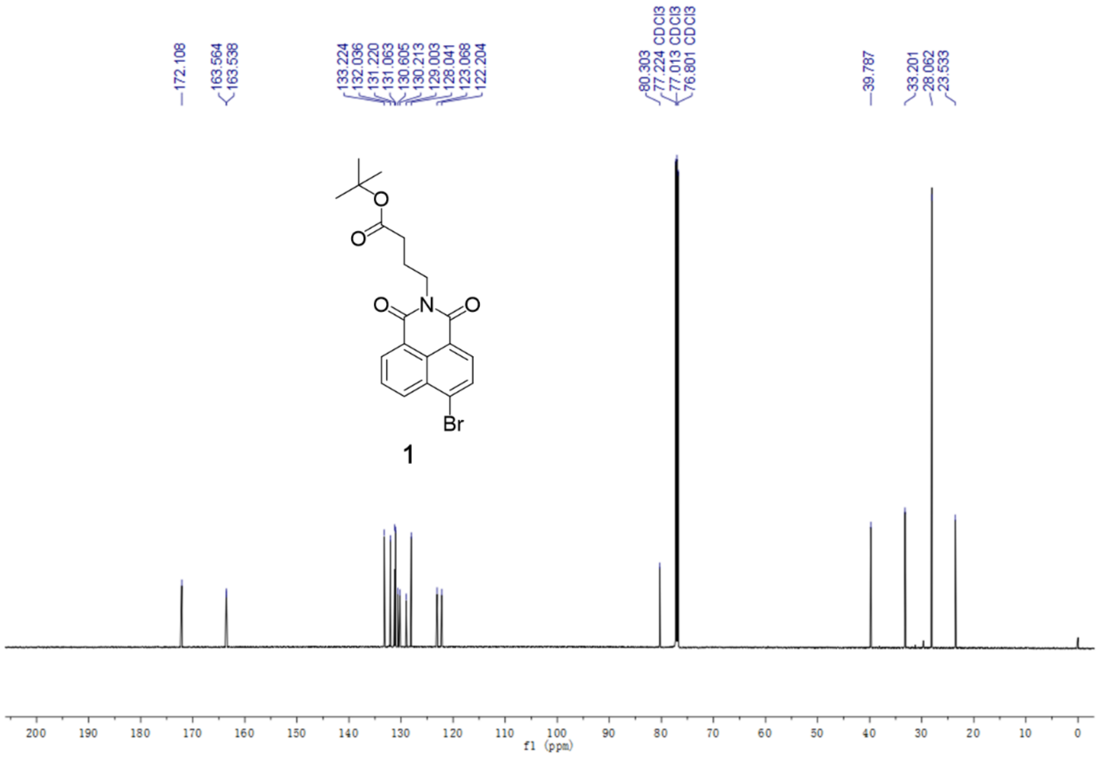

Figure S3. ^{13}C NMR spectra of compound **1**.

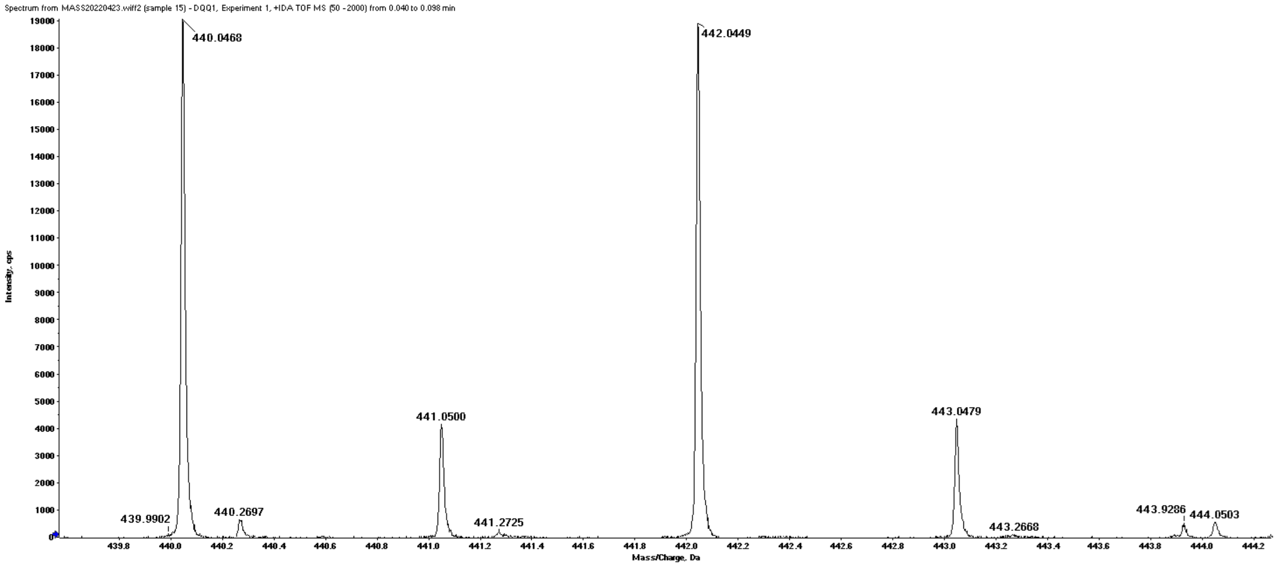

Figure S4. High-resolution mass spectra of compound **1.**

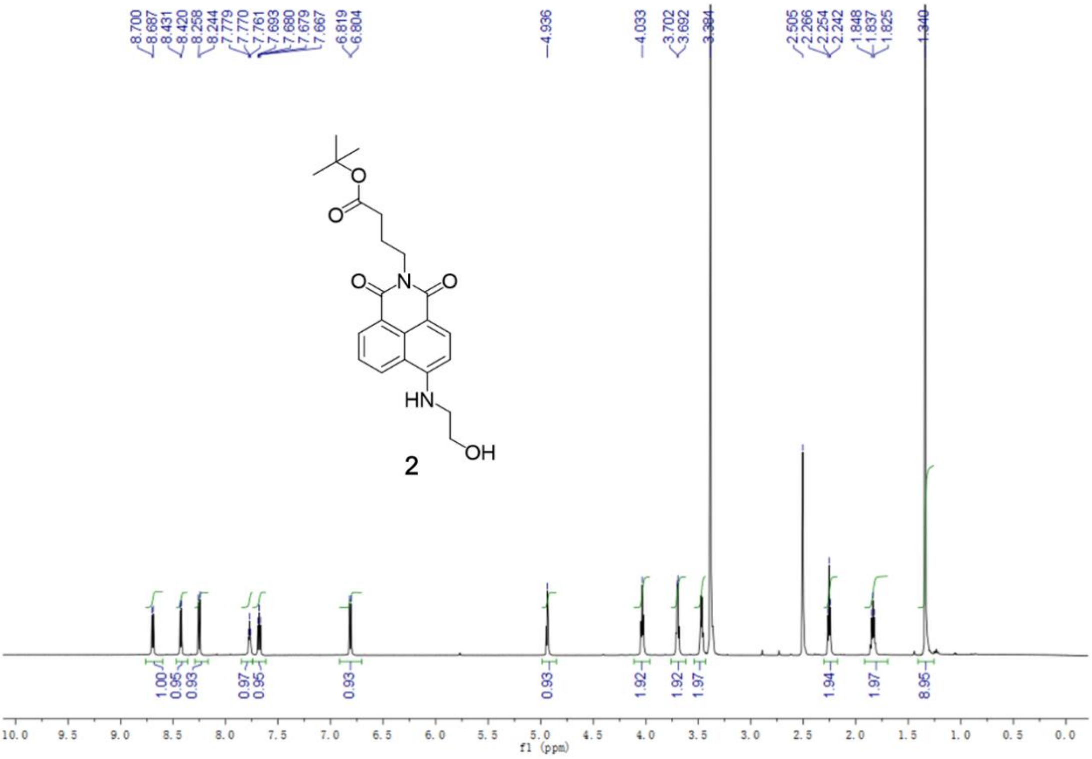

Figure S5. ^{1}H NMR spectra of compound **2**.

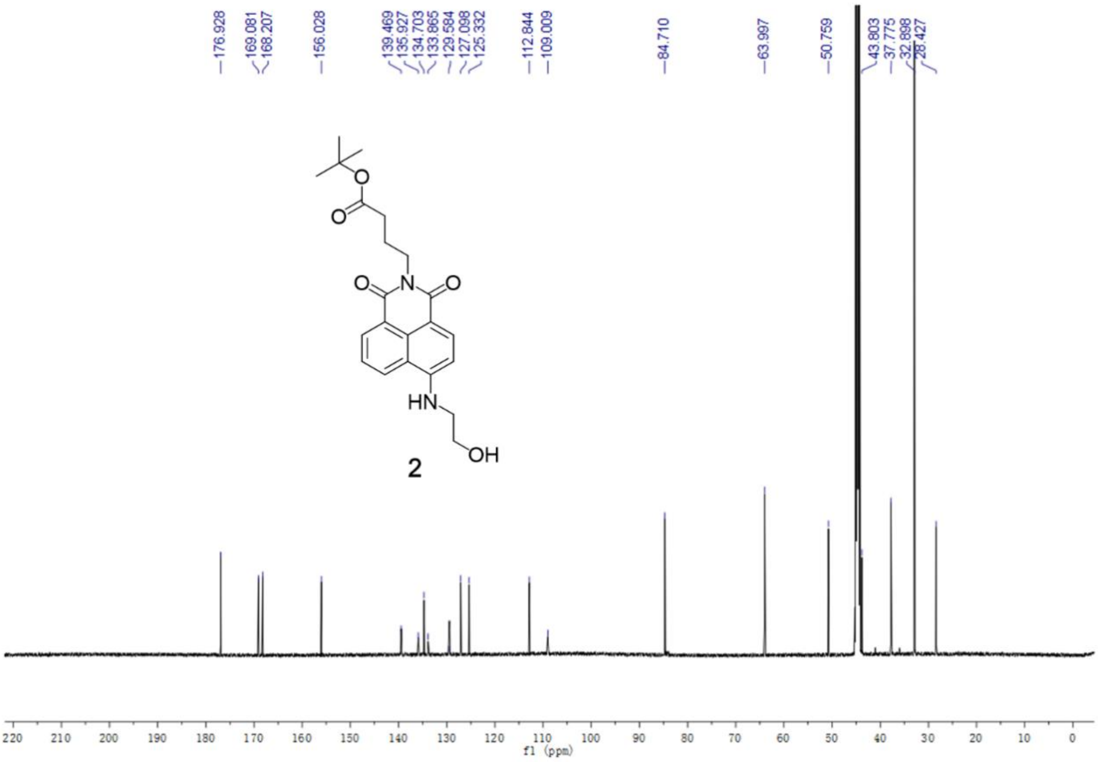

Figure S6. ^{13}C NMR spectra of compound **2**.

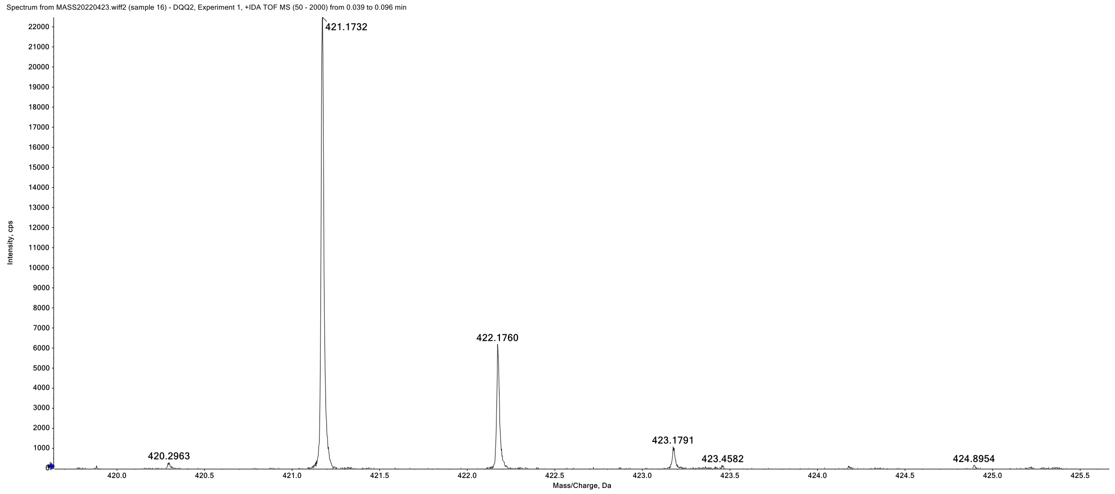

Figure S7. High-resolution mass spectra of compound **2**

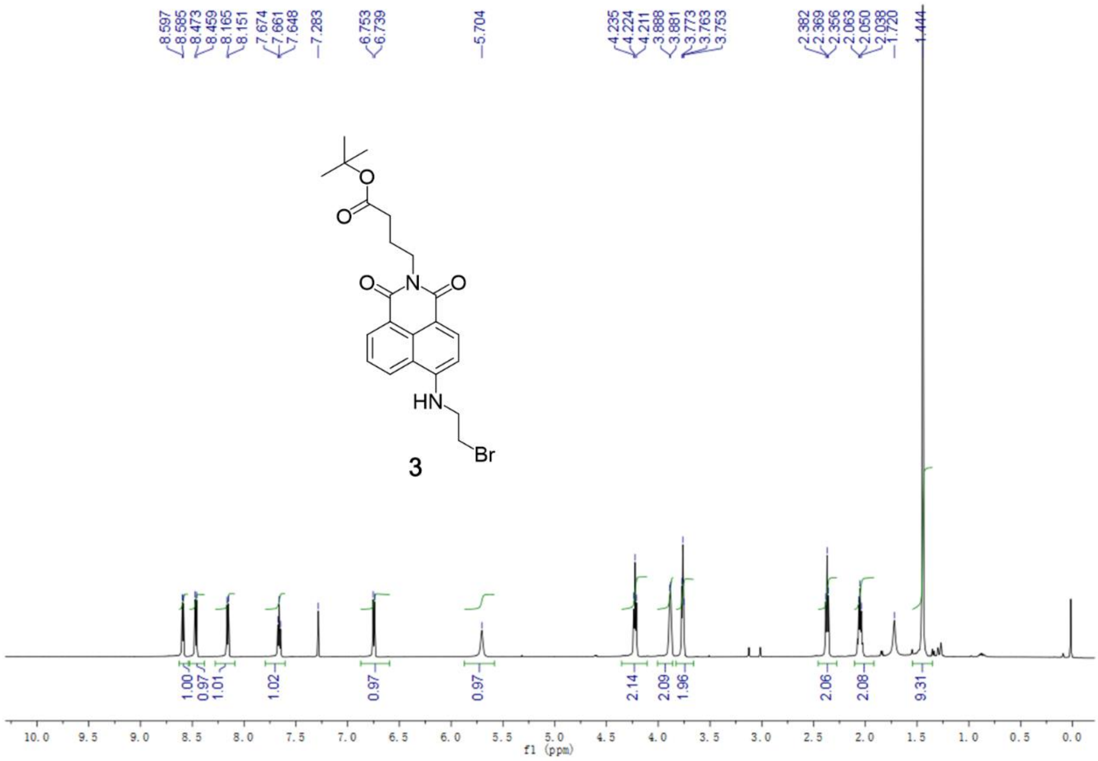

Figure S8. ^{1}H NMR spectra of compound **3**.

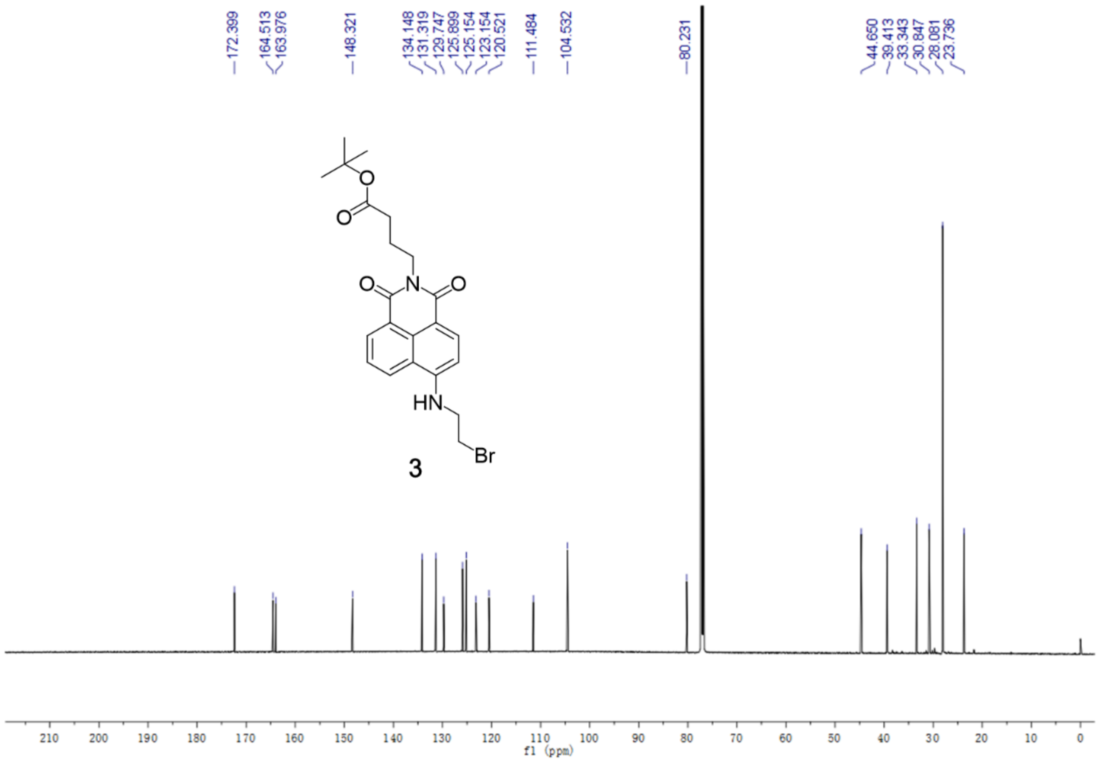

Figure S9. ^{13}C NMR spectra of compound **3**.

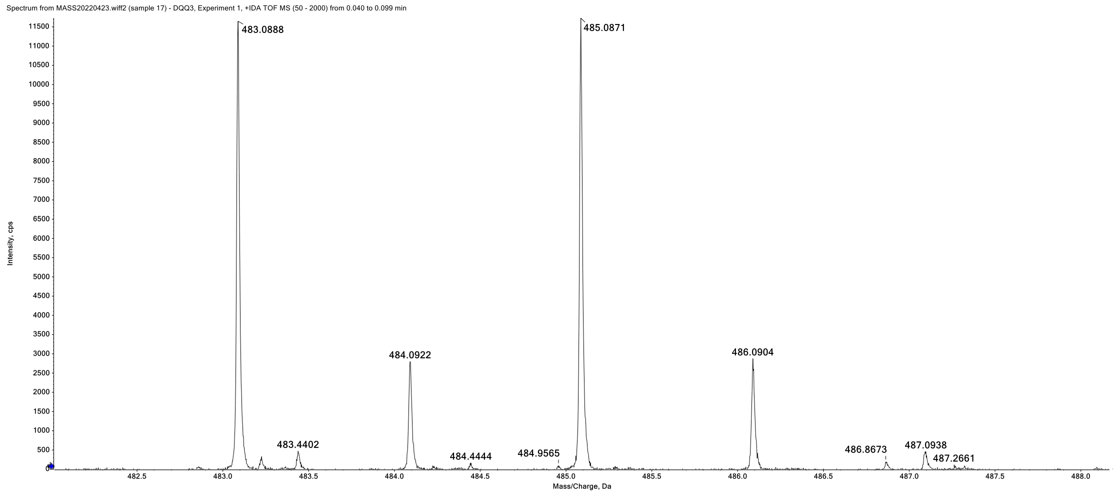

Figure S10. High-resolution mass spectra of compound **3**.

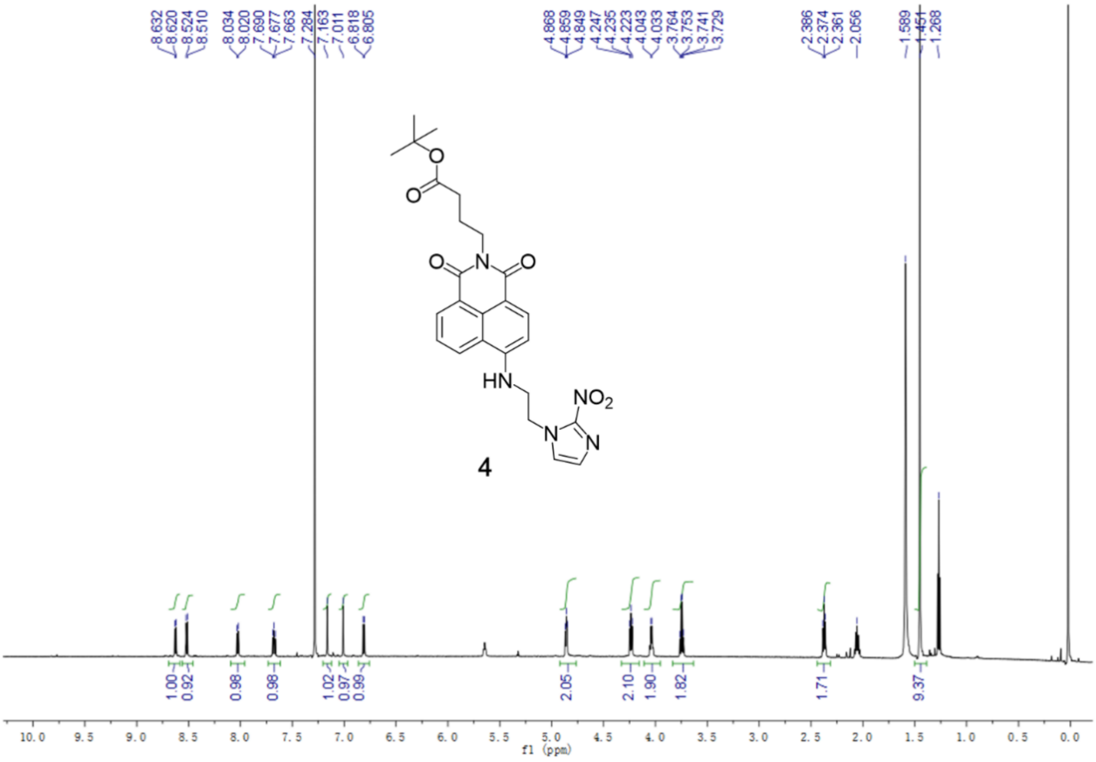

Figure S11. ^{1}H NMR spectra of compound **4**.

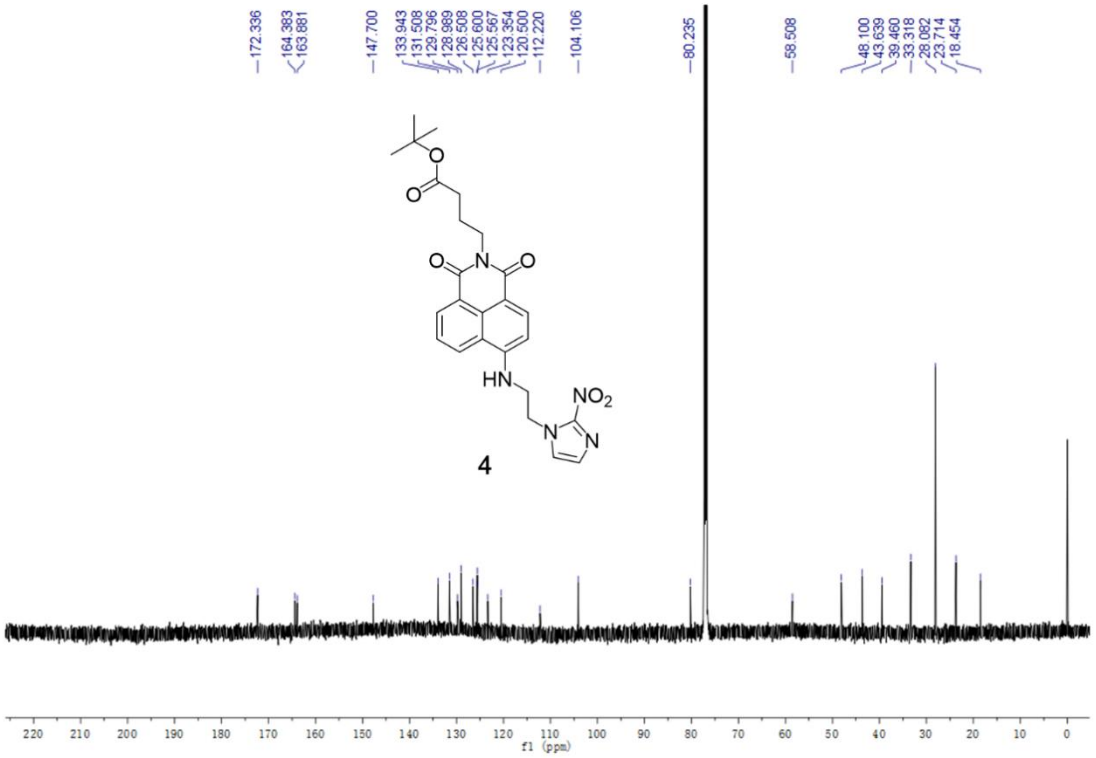

Figure S12. ^{13}C NMR spectra of compound **4**.

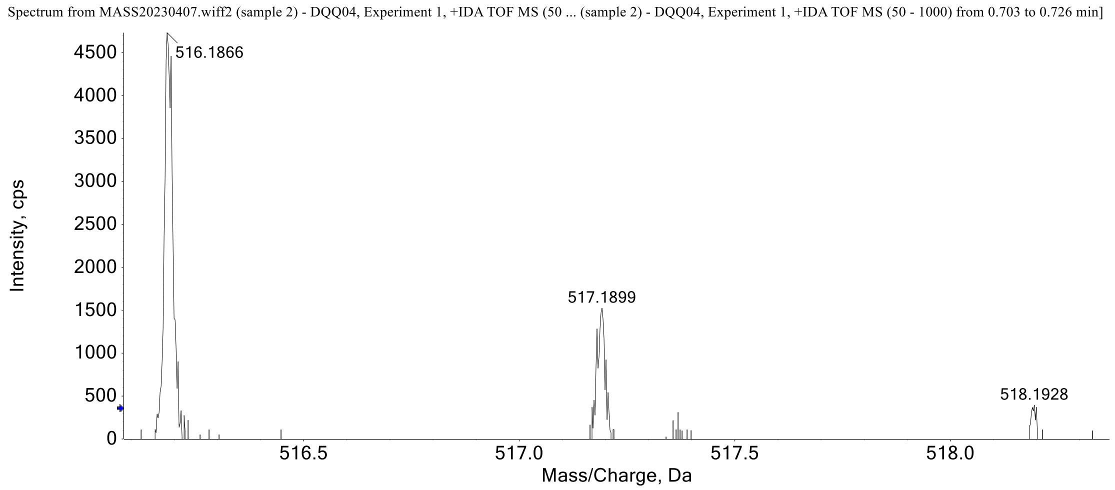

Figure S13. High-resolution mass spectra of compound **4**

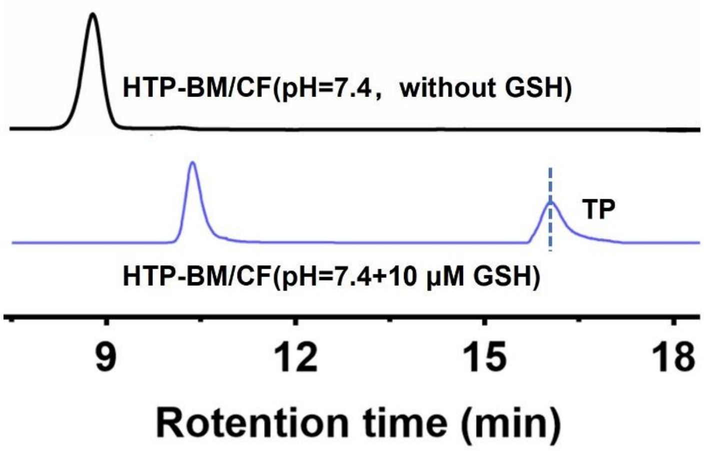

Figure S14. Catabolism of HTP-BM/CF at normal pH and high GSH concentrations

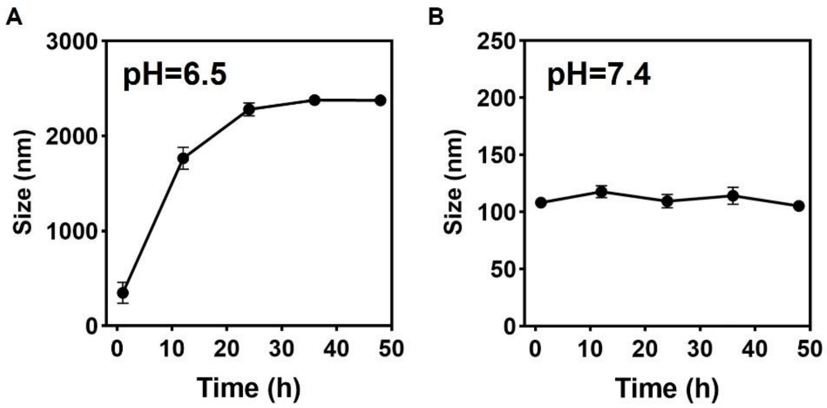

Figure S15. (A) The hydrodynamic size of HTP-BM/CFN at pH=6.5. (B) The hydrodynamic size of HTP-BM/CFN in 1640 medium with 10 % FBS.

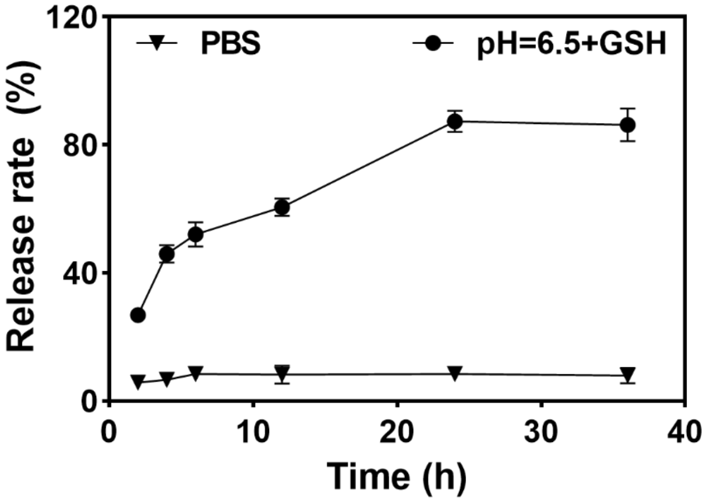

Figure S16. Release profiles of nanoparticles with time under different conditions.
